# CO-mediated restoration of mitochondrial redox homeostasis contributes to alleviating multiple programmed cell deaths in hepatic I/R injury

**DOI:** 10.1016/j.redox.2026.104440

**Published:** 2026-10-15

**Authors:** Jiayu Li, Mengzhu Xu, Qiang Zhou, Hanjing Ni, Chengge Shi, Tianci Zuo, Lingjun Zhang, Hongyang Lu, Wei He, Li Yu, Quazi T.H. Shubhra, Changlong Xu, Xiaojun Cai

**Affiliations:** a School and Hospital of Stomatology, Wenzhou Medical University, Wenzhou, 325027, China; b Department of Neurosurgery, Shanghai Tenth People's Hospital of Tongji University, Shanghai, 200072, China; c Department of Gastroenterology, the Second Affiliated Hospital and Yuying Children's Hospital of Wenzhou Medical University, Wenzhou, 325024, China; d Institute of Chemistry, University of Silesia in Katowice, Szkolna 9, Katowice, 40-006, Poland

**Keywords:** Hepatic ischemia–reperfusion injury, Controllable carbon monoxide delivery, Mitochondrial homeostasis, Ferroptosis, Cuproptosis, Disulfidptosis

## Abstract

Hepatic ischemia–reperfusion injury (HIRI) remains a life-threatening complication in liver surgery and transplantation with no clinically approved therapy. Here we develop a hyaluronic acid-modified, ROS-responsive nanoformulation (CPs@HA) that enables controllable delivery of carbon monoxide (CO) specifically to injured liver tissue. We show that mitochondrial dysfunction is closely associated with three distinct cell death pathways (ferroptosis, cuproptosis, and disulfidptosis) that occur in parallel during HIRI. CPs@HA restores mitochondrial homeostasis through activating the AMPK–SIRT1–PGC-1α axis and restoring Ca^2+^ balance, accompanied by suppressed mtROS burst, preserved mitochondrial membrane potential (ΔΨm), maintained cristae ultrastructure, and restored respiratory complex (I & IV) and PDHC activities. This dual restoration of ATP energy supply and NADPH reducing capacity, together with the direct inhibitory effect of CO on oxidative stress and inflammation, is associated with the attenuation of ferroptosis, cuproptosis, and disulfidptosis, consistent with transcriptomics and functional assays in both hepatic tissues and cellular models. Consequently, CPs@HA effectively attenuates liver injury. By interrupting the vicious cycle of oxidative stress, metabolic collapse, and cell death with mitochondrial repair as a key component, this work suggests a potentially unified therapeutic strategy against HIRI and indicates that targeting mitochondrial homeostasis may help mitigate multiple cell death programs in parallel.

## 1 Introduction

Hepatic ischemia–reperfusion injury (HIRI) remains a major complication of hepatectomy, liver transplantation and hemorrhagic shock, and is associated with early allograft dysfunction and poor long-term outcomes [1]. Clinical data show early allograft dysfunction in 53.6% of 621 liver transplant recipients, with a 90-day mortality of 16% and a 1-year graft survival of only 69.2% [2]. The hallmark of HIRI is the paradox that restoration of blood flow aggravates tissue damage, potentially leading to acute liver failure or multi-organ dysfunction [3], [4]. Despite decades of research, no effective therapy is clinically available, underscoring the urgent need for a deeper mechanistic understanding and novel interventions.

Oxidative stress is a central driver of HIRI pathology [5]. During ischemia, hypoxia impairs mitochondrial structure and ATP synthesis, while enhanced anaerobic metabolism generates reactive oxygen species (ROS), leaving mitochondria in a vulnerable “pre-activated state” [6]. Upon reperfusion, the sudden oxygen surge triggers a burst of ROS from the dysfunctional electron transport chain. Excessive ROS, together with intracellular Ca^2+^ overload, induces abnormal opening of the mitochondrial permeability transition pore (mPTP), causing collapse of the membrane potential, structural swelling and eventual rupture. Hence, mitochondrial damage in the reperfusion phase acts as a critical trigger for downstream injury cascades. Damaged mitochondria release mitochondrial DNA (mtDNA) and ROS, which directly injure cellular lipids, proteins, and DNA, and also release damage-associated molecular patterns (DAMPs) to activate innate immunity, fueling an inflammatory storm and a positive “oxidative stress–inflammation” loop [7], [8]. Within this hostile microenvironment, multiple oxidative stress-regulated programmed cell death pathways are activated in parallel, including ferroptosis, cuproptosis, disulfidptosis, and PANoptosis [9], [10], [11], [12]. Although these pathways have distinct execution mechanisms, they all converge on mitochondria as a common target or execution site, suggesting that mitochondrial dysfunction may represent a shared site of injury. However, whether targeting this mitochondrial hub can simultaneously suppress these diverse cell death pathways remains unexplored, and a systematic investigation into their concurrent regulation in HIRI is lacking.

Current clinical strategies fall short of addressing this complexity. Pharmacological agents such as N-acetylcysteine or glucocorticoids target single pathways and may increase infection risk and metabolic disorders [13]; ischemic preconditioning is invasive and difficult to apply in emergency or transplant settings [14]; and mechanical interventions like hypothermic machine perfusion are costly and technically demanding [6], [15]. Therefore, an intervention that restores mitochondrial homeostasis to simultaneously suppress multiple downstream cell death pathways is highly desired. Carbon monoxide (CO), an endogenous gaseous signaling molecule produced by heme oxygenase-1 (HO-1), has emerged as a promising candidate due to its antioxidant, anti-inflammatory, and mitochondrial-protective properties [16], [17]. CO activates the HO-1 pathway, downregulates ROS-related genes [18], [19], upregulates SIRT1, and suppresses NF-*κ*B-mediated inflammation [20], [21]. Consistent with these properties, previous studies showed that a CORM-401-loaded, hyaluronic acid-coated peptide dendrimer nanogel (CPHs) markedly attenuated oxidative stress and inflammation in experimental osteoarthritis [18]. In inflammatory bowel disease models, CORM-401-loaded nanogels (CMPs) and CO-releasing halloysite nanotubes (CMHs) similarly exerted potent antioxidant and anti-inflammatory effects, alleviating intestinal injury and restoring mucosal homeostasis [20], [22]. Beyond these antioxidant and anti-inflammatory effects, it also directly targets damaged mitochondria by reversibly binding to cytochrome *c* oxidase (Complex IV), inducing a controlled mitochondrial ROS signal that activates adaptive protective pathways and supports mitochondrial energy metabolism [23], [24]. Moreover, CO promotes mitophagy and supports mitochondrial biogenesis [17], [25]. Thus, CO not only reduces upstream oxidative and inflammatory insults but also help preserve mitochondrial homeostasis, thereby preserving mitochondrial redox and energy balance. We hypothesize that by restoring mitochondrial homeostasis, CO can interrupt the “oxidative stress–inflammation–cell death” cascade at a key upstream node, thereby simultaneously regulating multiple cell death pathways and improving HIRI outcomes. However, the clinical application of free CO is limited by its gaseous nature and lack of targeted delivery, necessitating a safe and controllable nanoformulation. Among the available CO donors, CORM-401 is a manganese carbonyl complex characterized by high CO-releasing capacity and oxidant-responsive release [26], [27]. These properties make CORM-401 particularly suitable for CO delivery in the ROS-rich microenvironment of HIRI. Although CO has demonstrated protective effects across diverse experimental liver injury models [28], [29], [30], [31], oxidant-responsive CORM-401 remains insufficiently investigated in HIRI. Based on this rationale, we constructed a multifunctional CO-releasing nanoformulation (CPs@HA) by loading the CO donor CORM 401 into poly(amidoamine) (PAMAM) nanogels and modifying the surface with hyaluronic acid (HA) (Scheme 1). The HA coating enhances serum stability and promotes CD44-mediated uptake by hepatic cells, contributing to the preferential accumulation and prolonged retention of CPs@HA in injured liver tissue. CPs@HA globally attenuates multiple oxidative stress-linked programmed cell death pathways, with ferroptosis, cuproptosis and disulfidptosis being the most significantly inhibited. Mechanistically, mitochondrial dysfunction is associated with changes of these pathways, while CPs@HA effectively restores mitochondrial homeostasis by activating the AMPK–SIRT1–PGC-1*α* axis and restoring Ca^2+^ balance, which inhibits mitochondrial ROS (mtROS) burst, preserves mitochondrial membrane potential (ΔΨm), maintains mitochondrial ultrastructure, recovers respiratory chain complex and PDHC activities, ATP synthesis and NADPH reducing capacity. By breaking the vicious cycle of oxidative stress, metabolic collapse and cell death, CPs@HA offers a unified therapeutic mechanism against HIRI. The novelty of this study lies in extending the investigation of CO-mediated hepatoprotection beyond its established antioxidant and anti-inflammatory effects to mitochondrial recovery and ferroptosis-, cuproptosis-, and disulfidptosis-associated processes in HIRI. Moreover, CPs@HA integrates improved CORM-401 stability, HA-mediated preferential delivery to injured liver tissue, and oxidant-responsive CO release, thereby linking controlled CO delivery with mechanistic investigation.

**Scheme 1:**
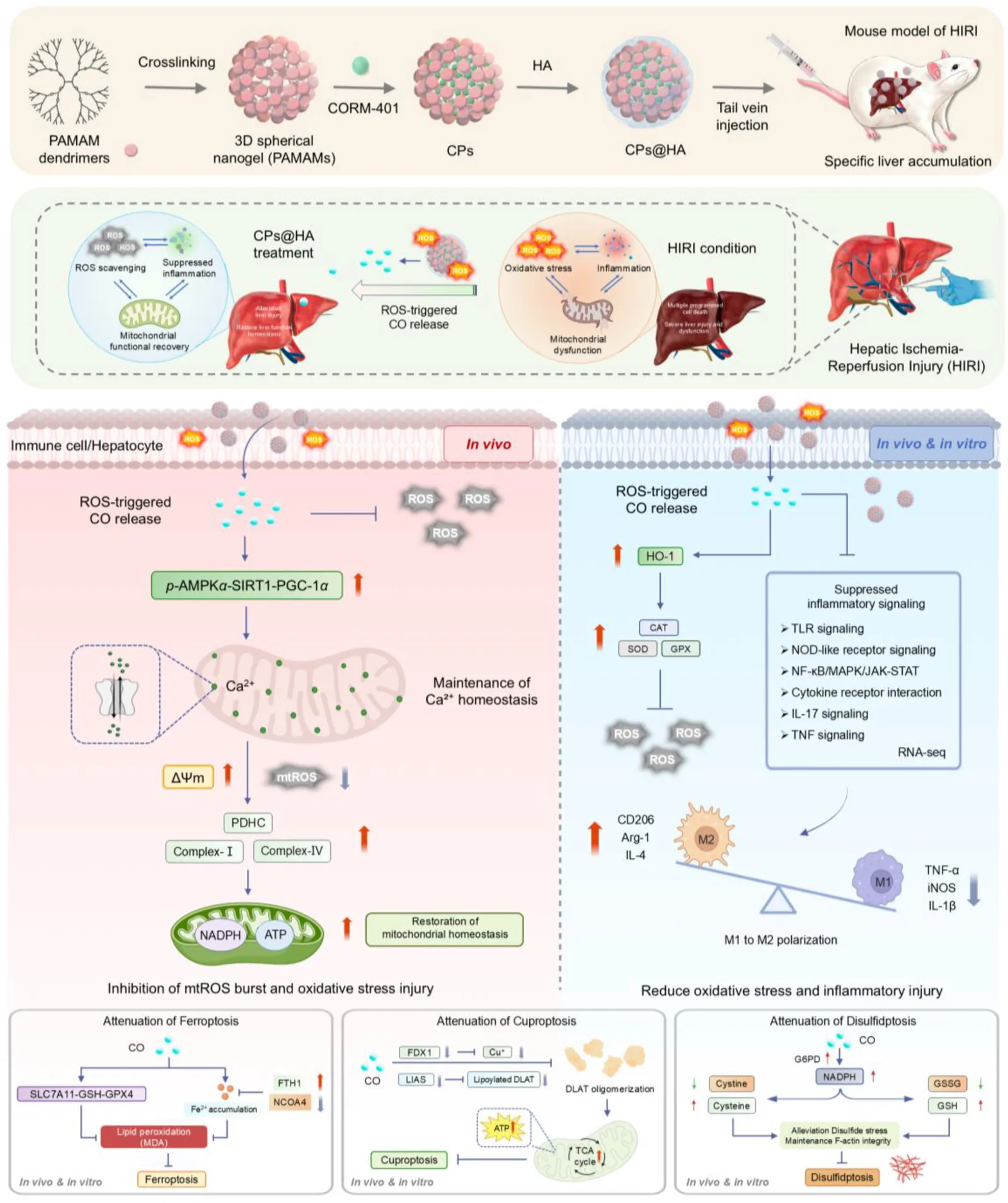
Schematic illustration of CPs@HA preparation and its proposed therapeutic mechanisms against HIRI: attenuation of oxidative stress and inflammation, restoration of mitochondrial homeostasis, and reduced ferroptosis, cuproptosis, and disulfidptosis. (1) CPs@HA restores mitochondrial homeostasis by activating the AMPK–SIRT1–PGC-1α axis and restoring Ca^2+^ balance. This leads to inhibition of mtROS burst, preservation of ΔΨm, and recovery of respiratory chain complex and PDHC activities, as well as restoration of ATP synthesis and NADPH reducing capacity. (2) CO promotes ROS clearance *via* HO-1 upregulation and enhanced CAT/SOD/GPX activities, reducing oxidative stress. It also suppresses inflammation by inhibiting TLR, NOD-like receptor, NF-κB/MAPK/JAK-STAT, TNF, and IL-17 pathways. (3) CO is associated with attenuated ferroptosis *via* the SLC7A11–GSH–GPX4 axis, accompanied by reduced Fe^2+^ accumulation and lipid peroxidation. (4) CO is associated with reduced cuproptosis through decreased Cu^+^ accumulation, inhibited DLAT oligomerization, and improved TCA cycle function. (5) CO is associated with reduced disulfidptosis through promotion of G6PD-dependent NADPH production, enhanced cystine-to-cysteine and GSSG-to-GSH reduction, restored Cys/GSH balance, and alleviated disulfide stress.

## 2 Results and discussion

### 2.1 Synthesis and characterization of CPs@HA

To prepare CPs@HA, we first crosslinked amino-terminated generation 3 PAMAM dendrimers with 3,3′-dithiodipropionic acid bis(N-succinimidyl ester) (DSP) to form stable nanogels (designated as PAMAMs). Subsequently, CORM-401 was encapsulated into the nanogels to obtain the drug delivery system CORM-401@PAMAMs (CPs) [32]. As shown in Fig. 1A, the prepared CPs appeared as a clear pale-yellow liquid. Dynamic light scattering (DLS) analysis revealed an average particle size of 205.9 nm, a zeta potential of +26.51 mV, and a polydispersity index (PDI) of 0.24, indicating a uniform size distribution. Given that the abundant amino groups on CPs readily adsorb serum proteins *via* electrostatic interactions, potentially leading to recognition and clearance by the reticuloendothelial system, we further modified CPs with HA to enhance *in vivo* circulation stability. HA coating increased the hydrophilicity of CPs, resulting in a larger hydrodynamic diameter of 252.5 nm and a slight rise in PDI (Fig. 1B). Concurrently, the zeta potential shifted from positive to negative (−21.95 mV) due to electrostatic interactions between the negatively charged carboxyl groups of HA and the amino groups on CPs, confirming successful HA modification. As shown in Fig. 1C and Supporting Information S1, CPs@HA exhibited good storage stability in PBS (pH 7.4), with no significant changes in particle size, PDI, or appearance after 7 days of incubation at 37 °C. More importantly, in serum stability tests (PBS containing 15% FBS, 37 °C), CPs@HA maintained stable particle size and zeta potential, demonstrating superior anti-protein adsorption capacity (Supporting Information Fig. S2 and S3). In contrast, CPs showed markedly inferior stability under the same conditions: particle size progressively increased with incubation time, while zeta potential gradually declined toward neutrality, indicative of serum protein adsorption and protein corona-induced aggregation [33]. These results indicate that HA modification confers a stable negative charge and a highly hydrated polysaccharide layer on CPs@HA, effectively inhibiting protein corona formation and significantly enhancing colloidal stability in physiological environments, which lays the material foundation for subsequent *in vivo* applications.

**Fig. 1:**
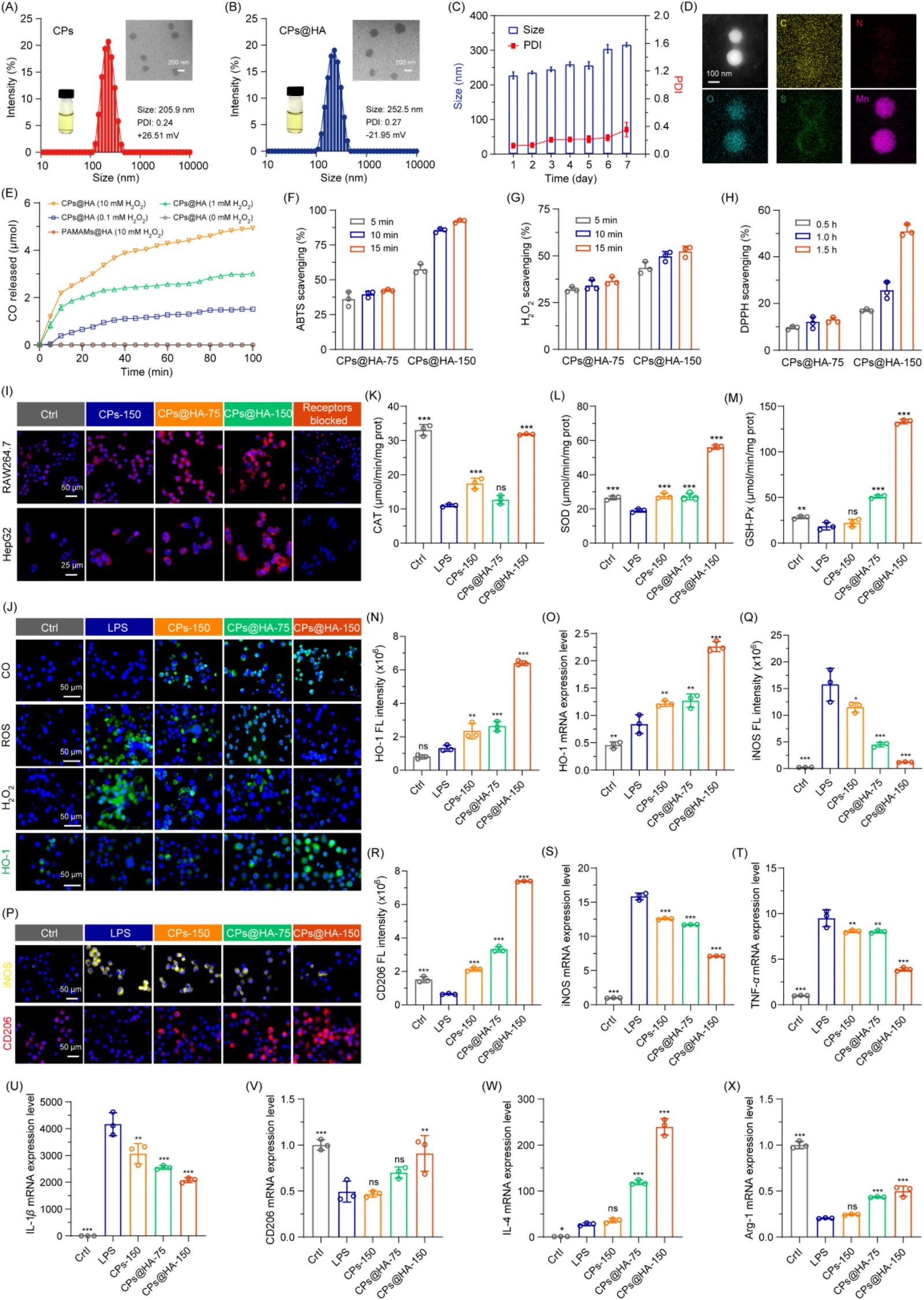
Physicochemical characterization, antioxidant, and anti-inflammatory activities of CPs@HA. (A, B) Size distribution, solution appearance, and representative TEM images (insets) of CPs and CPs@HA (scale bar = 200 nm). (C) Stability of CPs@HA during incubation at 37 °C for 7 days. (D) Elemental mapping of CPs@HA (scale bar = 100 nm). (E) CO release profiles of CPs@HA in the presence of different concentrations of H_2_O_2_. (F-H) Scavenging activities of CPs@HA against ABTS·^+^, H_2_O_2_, and DPPH·. (I) CLSM images showing the cellular uptake of Cy5.5-CPs and Cy5.5-CPs@HA by RAW264.7 and HepG2 cells (scale bar = 50 μm and 25 μm). (J) Fluorescence staining for intracellular CO generation, ROS and H_2_O_2_ scavenging, and IF staining for HO-1 expression in activated macrophages treated with CPs and CPs@HA (scale bar = 50 μm). (K-M) Activities of antioxidant enzymes (CAT, SOD, and GSH-Px) in activated macrophages treated with CPs and CPs@HA. (N) Quantitative analysis of IF intensity of HO-1. (O) mRNA expression level of HO-1. (P) IF staining for iNOS and CD206 in activated macrophages treated with CPs and CPs@HA (scale bar = 50 μm). (Q, R) Quantitative analysis of IF intensity of iNOS and CD206. (S-X) mRNA expression levels of iNOS, TNF-*α*, IL-1*β*, CD206, IL-4, and Arg-1. Statistical analyses were performed by comparing the LPS group with each of the other groups (*n* = 3). *P < 0.05, **P < 0.01, ***P < 0.001 *vs*. indicated. ns, not significant.

Transmission electron microscopy (TEM) images revealed that CPs@HA exhibited a clear spherical morphology (Fig. 1B, insets). Elemental mapping analysis showed that manganese (Mn), a characteristic element of CORM-401, was uniformly distributed within the CPs@HA nanogels without obvious local enrichment, further confirming homogeneous encapsulation of CORM-401 (Fig. 1D). UV–vis absorption spectroscopy revealed a characteristic absorption peak of CORM-401 at 370 nm for both CPs@HA and CPs (Supporting Information Fig. S4), confirming successful loading. The manganese (Mn) content of the samples was determined by inductively coupled plasma optical emission spectrometry (ICP-OES), from which the drug loading content (DLC) and loading efficiency (LE) of CORM-401 were calculated using a standard curve (Supporting Information Fig. S5). Under the optimized feeding ratio of CORM-401 to PAMAMs (1/1), CPs exhibited a DLC of 18.60% and an LE of 62% (Supporting Information Table S1). For CPs@HA prepared at a feeding ratio of CPs to HA (1/1), the Mn content was measured to be 2.88%; corresponding to a DLC of 14.45% and an LE of 57.8% (Supporting Information Table S2). In addition to quantifying loading, we examined the aqueous appearance of the formulation. CPs@HA and free CORM-401 released similar amounts of CO under 1 mM H_2_O_2_ (3.01 and 3.07 μmol, respectively) (Supporting Information Fig. S6).

To investigate the ROS-responsive CO release behavior of CORM-401 within CPs@HA, we monitored the release profile in real time using a CO detector in PBS containing various concentrations of H_2_O_2_ (0, 0.1, 1, and 10 mmol/L). As shown in Fig. 1E, after 100 min of incubation in H_2_O_2_-free PBS, almost no CO was detected, indicating good physiological stability of CPs@HA in the absence of ROS. As the H_2_O_2_ concentration increased from 0.1 mmol/L to 10 mmol/L, both the release rate and cumulative release increased in a concentration-dependent manner. After 100 min in 1 mmol/L and 10 mmol/L H_2_O_2_, the cumulative CO release reached 2.98 μmol and 4.83 μmol, respectively, demonstrating the high responsiveness of CPs@HA to H_2_O_2_ and its oxidation-triggered CO release under pathological conditions. As a negative control, PAMAMs@HA (without CORM-401 loading) showed no CO release even under 10 mmol/L H_2_O_2_, ruling out non-specific CO release from the carrier and confirming that the released CO originated exclusively from the responsive degradation of CORM-401. The CO-depleted control, iCPs@HA retained a particle size (258.6 nm) and zeta potential (−26.65 mV) close to those of CPs@HA (Supporting Information Fig. S7). Collectively, CPs@HA exhibits ROS-triggered on-demand CO release, enabling selective CO release in oxidative stress microenvironments while maintaining stability under physiological conditions. This release profile is consistent with previous reports identifying CORM-401 as an oxidant-sensitive CO donor whose CO release is enhanced by biologically relevant oxidants, particularly H_2_O_2_, in a concentration-dependent manner [26], [34]. CORM-401 also reduced intracellular ROS accumulation and cell death, supporting its protective activity under oxidative stress [27]. Therefore, CPs@HA is particularly well suited to the ROS-rich microenvironment of HIRI. This property provides a crucial safety foundation for subsequent *in vivo* applications. After verifying its ROS-responsive CO release behavior, we next evaluated the *in vitro* antioxidant activity of CPs@HA using three classical free radical scavenging systems: ABTS·^+^, H_2_O_2_, and DPPH·. As shown in Fig. 1F–H, CPs@HA exhibited significant time- and concentration-dependent scavenging effects against all three radicals. At the optimal concentration of 150 μg/mL, CPs@HA achieved the highest scavenging activity. In the ABTS·^+^ system, it rapidly reached a 92.84% scavenging rate within 15 min, approaching near-complete clearance. In the H_2_O_2_ system, a 52.37% scavenging rate was achieved within the same timeframe. In contrast, the DPPH· system exhibited significantly slower kinetics, with the scavenging rate for the 150 μg/mL group ranging from 16.56% to 50.85% over 0.5–1.5 h. This difference is likely attributable to the assay conditions: DPPH· assays are typically conducted in organic solvents (e.g., methanol), in which the highly hydrophilic CPs@HA disperses poorly, thus requiring a longer reaction time to reach equilibrium [35].

To determine safe concentrations for subsequent experiments, we first evaluated the biosafety of CPs@HA. Comparative studies with unmodified CPs demonstrated that CPs@HA, owing to the protective HA coating, exhibited excellent cytocompatibility and hemocompatibility. Cytotoxicity assays revealed that CPs moderately reduced RAW264.7 cell viability at 120 μg/mL, as evidenced by the presence of some PI-labeled dead cells and a viability of 86% (Supporting Information Fig. S8 and S9). This is likely due to electrostatic interactions between the positively charged nanogel surface and negatively charged cell membrane phospholipids, leading to membrane perturbation and cellular stress [36]. In contrast, CPs@HA showed markedly improved cytocompatibility at the same concentration, with a mild increase in red fluorescence observed only at concentrations above 200 μg/mL, and cell viability remained >95% at concentrations ≤150 μg/mL. A similar trend was confirmed in HepG2 cells, where almost no PI-labeled dead cells were observed and cell viability remained >95% at CPs@HA concentrations ≤150 μg/mL (Supporting Information Fig. S10 and S11). Hemocompatibility was further validated by hemolysis tests, with CPs@HA exhibiting a hemolysis rate <5% even at 150 μg/mL (Supporting Information Fig. S12). Based on these results, we selected 75 μg/mL and 150 μg/mL as the test concentrations for subsequent studies, ensuring cellular safety while maintaining an appropriate dose gradient for functional analyses.

Efficient cellular uptake is a prerequisite for CPs@HA to exert therapeutic effects in HIRI. This pathological process involves two key cell types: activated hepatic macrophages (Kupffer cells), which mediate inflammation and oxidative stress, and hepatocytes, which are the primary targets of oxidative stress injury [37]. Given that CD44 is a key receptor for HA and is expressed on both activated macrophages and hepatocytes [38], we employed HA as a targeting ligand to construct CPs@HA for simultaneous recognition of these cell types. To verify dual-targeting ability, we conducted uptake experiments using lipopolysaccharide (LPS)-activated RAW264.7 macrophages (simulating Kupffer cells) and HepG2 hepatocytes. HepG2 cells were selected because they are a well-established human hepatic cell line widely used in studies of liver injury and oxidative stress [39]. Unlike commonly used hepatic cell lines that have been reported to be cross-contaminated, such as L-02/HL-7702 and WRL-68, (both identified as HeLa-contaminated) [40], HepG2 cells are considered more reliable. In addition, unlike primary hepatocytes, which rapidly lose liver-specific functions and undergo dedifferentiation under conventional culture conditions [41], HepG2 cells offer a more practical *in vitro* model for routine uptake studies. As shown in Fig. 1I, after incubation with Cy5.5-labeled nanogels, LPS-stimulated activated macrophages treated with Cy5.5-CPs@HA exhibited strong dose-dependent red fluorescence, confirming efficient HA-mediated uptake. In contrast, the signal from Cy5.5-CPs (120 μg/mL) was markedly weaker, indicating limited uptake *via* non-specific phagocytosis. Free HA competition further confirmed that the enhanced uptake of CPs@HA resulted from specific recognition between surface HA and CD44 receptors on macrophages [42]. CPs@HA also demonstrated efficient dose-dependent uptake in HepG2 cells, which was competitively inhibited by free HA, verifying its targeting ability for CD44-expressing hepatocytes. Quantitative analysis using ImageJ software corroborated these observations (Supporting Information Fig. S13 and S14). In summary, CPs@HA achieves CD44-mediated dual targeting of activated macrophages and hepatocytes, enabling efficient delivery of CORM-401 to the HIRI lesion site.

Given the critical role of oxidative stress in HIRI, we systematically evaluated the antioxidant performance of CPs@HA. First, we verified its CO release capability within activated macrophages. As shown in Fig. 1J and Supporting Information S15, CPs@HA-150 treatment significantly enhanced the CO probe signal, indicating effective intracellular CO release. We next investigated its ability to scavenge ROS. LPS stimulation markedly increased ROS and H_2_O_2_ fluorescence signals in activated macrophages (Fig. 1J and Supporting Information S16 and S17), indicating oxidative stress accumulation. While CPs treatment showed limited scavenging efficacy, CPs@HA-150 treatment nearly restored ROS and H_2_O_2_ levels to those of the control group, effectively alleviating inflammation-induced oxidative stress. We further examined the activities of three key antioxidant enzymes: CAT, SOD, and GSH-Px. CPs@HA-150 treatment significantly enhanced the activities of all three enzymes, with CAT activity restored to near-control levels, while SOD and GSH-Px activities exceeding those in other treatment groups (Fig. 1K–M). Given that these enzymes are downstream targets of the Nrf2–HO-1 pathway, we next investigated the expression of HO-1. CPs@HA-150 treatment significantly enhanced HO-1 fluorescence (Fig. 1J and N). qRT-PCR further confirmed a 2.26-fold increase in HO-1 mRNA levels (Fig. 1O). These results indicate that CPs@HA not only reduces oxidative stress but also activates HO-1-related signaling pathways.

Building on the verified antioxidant performance of CPs@HA, we next assessed its anti-inflammatory effects. First, we evaluated morphological changes in activated RAW264.7 cells. Normal cells exhibited a uniform, rounded morphology and were mildly adherent. Following LPS induction, cells displayed typical M1 phenotypic changes, including increased spreading, enhanced pseudopodia formation, and marked morphological flattening (Supporting Information Fig. S18). In contrast, CPs@HA-150 treatment restored cells to a relatively rounded or elongated spindle-shaped morphology. Quantitative analysis confirmed that CPs@HA-150 significantly increased the maximum elongation rate from 0.62 (LPS group) to 1.96 and reduced the number of cell protrusions to 0.41-fold of the LPS group (Supporting Information Fig. S19 and S20), demonstrating effective inhibition of LPS-induced morphological activation. Immunofluorescence (IF) staining further verified macrophage polarization (Fig. 1P–R). The LPS group showed significantly enhanced iNOS (M1 marker) signals but weak CD206 (M2 marker) signals, indicating M1 predominance. Following CPs@HA-150 treatment, iNOS signals were markedly diminished (to 0.08-fold of the LPS group) while CD206 signals were significantly enhanced (increased by 16.42-fold), suggesting an increased M2 macrophage proportion. qRT-PCR results corroborated these observations (Fig. 1S–X). LPS stimulation significantly upregulated the pro-inflammatory factors iNOS (15.85-fold), TNF-*α* (9.77-fold), and IL-1*β* (4169.17-fold) compared to the normal group. CPs@HA-150 treatment most effectively reversed this upregulation, reducing iNOS, TNF-*α*, and IL-1*β* expression to 0.45-fold, 0.41-fold, and 0.50-fold of the LPS group levels, respectively. Importantly, CPs@HA-150 significantly increased anti-inflammatory factors: CD206 (1.86-fold), IL-4 (8.57-fold), and Arg-1 (2.50-fold) compared to the LPS group. Together, these results demonstrate that CPs@HA-150 effectively inhibits pro-inflammatory macrophage polarization and promotes conversion to an anti-inflammatory phenotype, reshaping the inflammatory microenvironment at both protein and transcriptional levels. Collectively, CPs@HA-150 exhibits potent antioxidant and anti-inflammatory activities, which are attributed to its ability to scavenge ROS, restore antioxidant enzyme activities, upregulate HO-1 expression, and modulate macrophage polarization.

### 2.2 Therapeutic efficacy of CPs@HA in a mouse model of HIRI

Based on the excellent antioxidant and anti-inflammatory activities of CPs@HA *in vitro*, we next validated its *in vivo* therapeutic efficacy in a mouse model of HIRI. HIRI was induced by clamping the portal triad for 1 h followed by reperfusion [43]. To determine the optimal therapeutic dose, mice received tail vein injections of CPs@HA (0.25–2 mg/mL) 2 h before ischemia and were euthanized 24 h post-reperfusion for assessment of liver function (aspartate aminotransferase, AST; alanine aminotransferase, ALT) and COHb levels. As shown in Supporting Information Fig. S21A and B, AST and ALT levels declined in a dose-dependent manner, with the maximal reduction observed at 1.5 mg/mL (AST: 211.8 U/L; ALT: 102.5 U/L). No further improvement was observed at 2 mg/mL, indicating a plateau effect. Importantly, COHb levels remained within the normal range across all doses (Fig. S21C), confirming the absence of systemic CO toxicity. Based on these efficacy and safety profiles, 1.5 mg/mL was selected as the optimal dose for subsequent studies, balancing potent hepatoprotection with minimal off-target effects.

Using the optimal dose identified above, we systematically evaluated the *in vivo* therapeutic efficacy of CPs@HA (Fig. 2A). The treatment schedule, sample collection, and principal analyses are summarized in Supporting Information Fig. S22. Experimental animals were randomly divided into seven groups: (1) Normal, (2) Sham-operated, (3) HIRI model, (4) PAMAMs@HA (1.5 mg/mL) + HIRI, (5) CPs (1.2 mg/mL) + HIRI, (6) CPs@HA (1.5 mg/mL) + HIRI, and (7) iCPs@HA (1.5 mg/mL) + HIRI. PAMAMs@HA served as the blank nanogel control without CORM-401 loading, while iCPs@HA served as the inactivated control with pre-released CO. All treatment groups received the same administration regimen as described in the dose-finding experiment. Mice were euthanized 24 h after reperfusion, and blood and liver tissue samples were collected for serum biochemical analysis, histopathological examination, and molecular assays to systematically assess liver injury and the protective effects of each treatment.

**Fig. 2:**
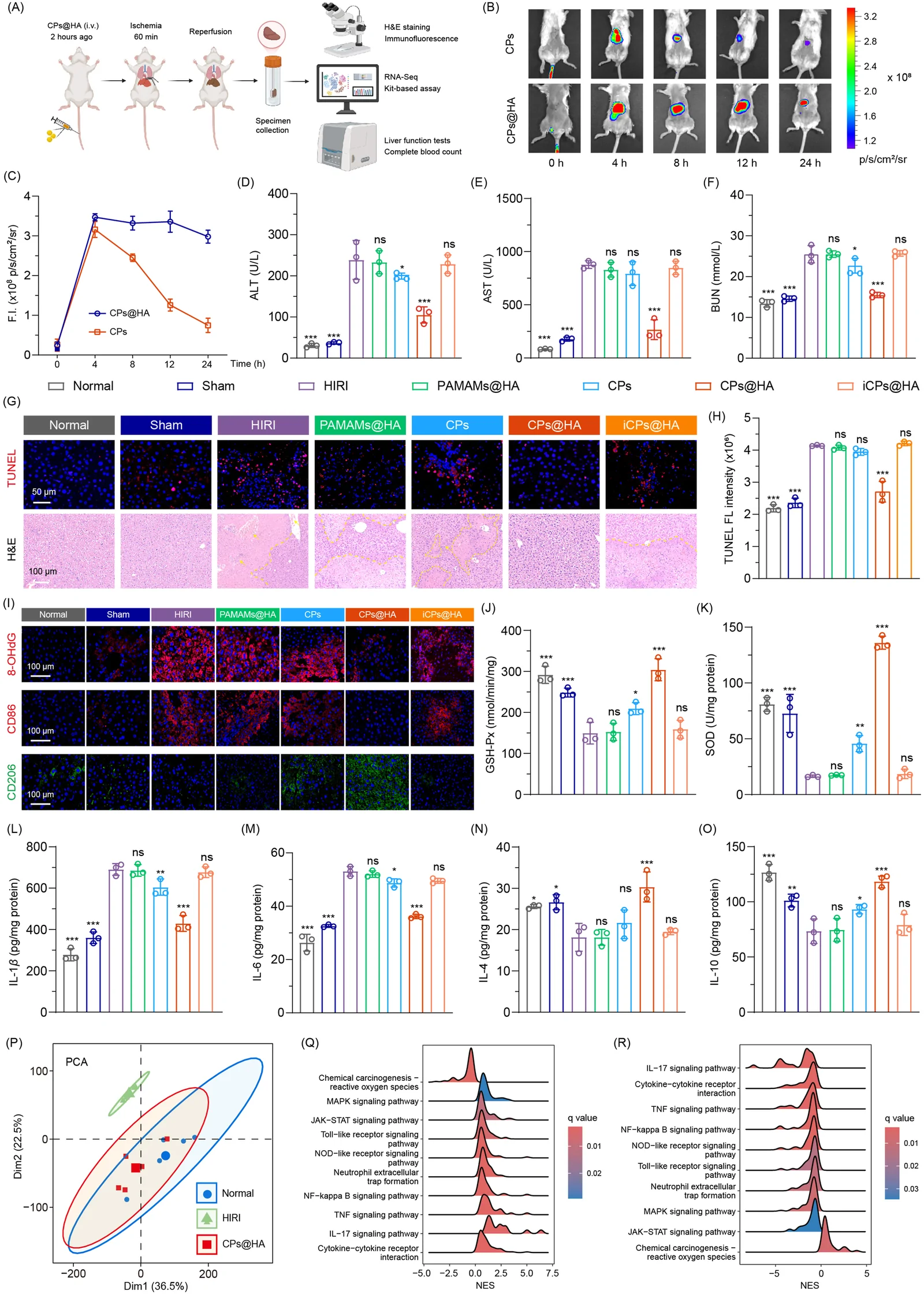
*In vivo* therapeutic evaluation of CPs@HA in HIRI. (A) Schematic illustration of the experimental timeline for the HIRI model (created with BioRender.com). (B) *In vivo* fluorescence imaging of Cy5.5-CPs and Cy5.5-CPs@HA, showing liver accumulation at different time points after intravenous injection. (C) Quantitative analysis of liver fluorescence intensity at 0, 4, 8, 12, and 24 h post-injection. (D-F) Serum levels of ALT, AST, and BUN in HIRI mice. (G) Representative images of TUNEL and H&E staining of liver tissues from different treatment groups (scale bar = 50 μm and 100 μm). (H) Quantification of TUNEL-positive area. (I) IF staining for the oxidative DNA damage marker 8-OHdG, the M1-type macrophage marker CD86, and the M2-type macrophage marker CD206 in liver sections (scale bar = 100 μm). (J, K) Activities of GSH-Px and SOD in liver tissues. (L-O) Inflammatory cytokine expression levels (IL-1*β*, IL-6, IL-4, and IL-10) in liver tissues determined by ELISA. (P) PCA of samples from the Normal, HIRI and CPs@HA groups. (Q) GSEA comparing the Normal and HIRI groups. (R) GSEA comparing the HIRI and CPs@HA groups. Statistical analyses were performed by comparing the HIRI group with each of the other groups (*n* = 3). *P < 0.05, **P < 0.01, ***P < 0.001 *vs*. indicated. ns, not significant.

Before evaluating therapeutic efficacy, we investigated the liver-targeting capability of CPs@HA in HIRI mice using an *in vivo* imaging system (IVIS). As shown in Fig. 2B and C, Cy5.5-CPs@HA exhibited persistent fluorescence signals in the liver for up to 24 h after tail vein injection, whereas signals from Cy5.5-CPs decayed rapidly, falling to background levels within approximately 12 h. At 24 h post-injection, ex vivo fluorescence imaging was performed on major organs, including the heart, liver, spleen, lung, and kidney. These results further showed that the fluorescence signal in the liver of HIRI mice in the CPs@HA group was markedly higher than that in other organs, indicating pronounced liver accumulation (Supporting Information Fig. S23). This difference in biodistribution stems from distinct surface properties. Positively charged CPs readily adsorb plasma proteins, forming a protein corona that accelerates Kupffer cell-mediated clearance [44]. In contrast, HA modification confers a negatively charged, hydrophilic surface that reduces protein adsorption and delays clearance [45]. Additionally, HA enhances retention in injured livers through (1) electrostatic adhesion to positively charged components in the inflamed microenvironment, such as extracellular histones [46], and (2) specific binding to upregulated CD44 receptors [47]. To verify responsive CO release in the injured liver microenvironment, we performed CO probe staining on frozen liver sections. As shown in Supporting Information Fig. S24 and S25, the CPs@HA group exhibited the strongest CO fluorescence signal, significantly higher than that of the CPs and other control groups, further confirming that HA modification enhances targeted delivery and promotes *in situ* CO release. The iCPs@HA group showed almost no fluorescence, confirming that the signal originated from CO released by CORM-401.

To evaluate the hepatoprotective effects of different treatments, we first examined changes in liver and kidney function using serum biochemical parameters. The HIRI group showed significantly elevated serum ALT and AST activities (Fig. 2D and E), indicating severe hepatocyte injury. Blood urea nitrogen (BUN) levels were also markedly increased (Fig. 2F), suggesting secondary renal impairment and reduced clearance of nitrogenous metabolites following HIRI [48]. Compared with the HIRI group, CPs@HA treatment significantly reduced ALT, AST, and BUN levels to 44%, 30%, and 61% of HIRI levels, respectively, approaching normal values. In contrast, the other treatment groups showed limited improvement. The CPs group achieved only partial reduction, while the PAMAMs@HA and iCPs@HA groups maintained elevated levels across all indicators.

We further assessed hepatocyte apoptosis using TUNEL staining (Fig. 2G and H). The HIRI group exhibited numerous TUNEL-positive cells compared with the Normal and Sham groups, indicating intense DNA fragmentation-related cell death. Notably, the CPs@HA group showed a marked reduction in positive cells, demonstrating a pronounced anti-apoptotic effect. In contrast, the PAMAMs@HA and iCPs@HA groups showed strong positive signals comparable to the HIRI group, while the CPs group achieved only partial reduction. H&E staining further corroborated these findings at the tissue morphology level (Fig. 2G). Liver tissues from the HIRI, PAMAMs@HA, and iCPs@HA groups all exhibited pronounced damage characteristics, including hepatocyte degeneration, focal necrosis-like changes, disordered hepatic sinusoid structure, and dense inflammatory cell infiltration. The CPs group partially alleviated these pathological changes, albeit with limited improvement. In contrast, the CPs@HA group showed the most well-preserved hepatic lobular structure, with significantly reduced necrosis and inflammatory infiltration, demonstrating optimal restoration of tissue morphology. In summary, CPs@HA, through HA-mediated targeted delivery and lesion-specific CO release, exhibited optimal protective effects in improving liver and kidney function, inhibiting apoptosis, and maintaining tissue structural integrity.

In addition to its excellent hepatoprotective effects, CPs@HA demonstrated outstanding biocompatibility. First, CPs@HA exhibited excellent hematological safety. It did not induce systemic CO toxicity, as plasma COHb levels remained stable within 24 h following administration (Supporting Information Fig. S26). Key hematological indicators, including hemoglobin, red blood cells, and platelets, remained within normal ranges (Supporting Information Fig. S27), indicating no significant hemolysis, bone marrow suppression, or coagulation abnormalities at the tested dose. Additionally, while white blood cell and neutrophil counts were elevated in the HIRI group, consistent with a systemic inflammatory response, these levels were significantly reduced in the CPs@HA group, suggesting effective inflammation mitigation without additional toxic burden. Histological evaluation of major organs (heart, liver, spleen, lung, kidney) further confirmed the safety of CPs@HA. As shown in Supporting Information Fig. S28, tissues remained well-preserved, with regularly arranged myocardial fibers, intact liver structure, clear splenic white/red pulp boundaries, normal alveolar architecture, and unremarkable renal histology. In conclusion, CPs@HA achieves effective HIRI intervention while ensuring biosafety, providing a reliable foundation for subsequent efficacy and mechanistic studies.

Given that oxidative stress is a key driver of HIRI pathogenesis, we next assessed oxidative damage and endogenous antioxidant defenses at the tissue level. As shown in Fig. 2I and Supporting Information S29, 8-OHdG IF signals were significantly enhanced in the HIRI group, indicating aggravated oxidative DNA damage after reperfusion [49]. Concurrently, the activities of GSH-Px, SOD, and CAT were significantly reduced (Fig. 2J and K and Supporting Information S30), reflecting impairment of the endogenous antioxidant system. Compared with the HIRI group, CPs treatment showed limited improvement, whereas CPs@HA significantly reduced 8-OHdG levels (to 0.35-fold of HIRI levels) and restored GSH-Px, SOD, and CAT activities (increased to 2.03-fold, 8.24-fold, and 1.79-fold of HIRI levels, respectively), demonstrating its ability to alleviate oxidative damage and restore antioxidant function. Given that ROS burst during HIRI can amplify inflammatory cascades, we further validated the protective mechanism of CPs@HA from the perspective of inflammatory regulation. IF staining results (Fig. 2I and Supporting Information S31 and S32) showed that Kupffer cells in the HIRI group exhibited pronounced pro-inflammatory phenotypic characteristics, including enhanced CD86 signals and weakened CD206 signals, indicating macrophage polarization toward the M1 type [50]. In contrast, CPs@HA treatment significantly reversed this trend, with CD86 signals markedly reduced and CD206 signals significantly enhanced, demonstrating its ability to suppress pro-inflammatory responses and promote conversion to the reparative M2 phenotype. This histological evidence was highly consistent with changes in inflammatory factor expression. Pro-inflammatory factors IL-1*β* and IL-6 were significantly elevated in the HIRI group (Fig. 2L and M), while CPs@HA treatment reduced them to 0.62-fold and 0.68-fold of HIRI levels, respectively. Simultaneously, anti-inflammatory factors IL-4 and IL-10 were significantly upregulated in the CPs@HA group (Fig. 2N and O), increasing to 1.67-fold and 1.53-fold of HIRI levels, respectively, approaching levels in the Normal and Sham groups. In summary, CPs@HA exhibits comprehensive effects in regulating macrophage phenotype and reshaping the inflammatory factor network under HIRI conditions, with its anti-inflammatory effects synergizing with antioxidant protection to collectively alleviate reperfusion injury.

The above results demonstrate that CPs@HA exhibits consistent protective effects across serum biochemical indicators, histological damage, and oxidative stress- and inflammation-related phenotypes. To further elucidate its underlying molecular mechanisms, we performed RNA-seq on liver tissues from normal, HIRI, and CPs@HA-treated mice. PCA revealed clear separation between HIRI and normal samples (Fig. 2P), indicating significant remodeling of global gene expression profiles after IR. In contrast, CPs@HA-treated samples clustered closer to the normal group, suggesting effective reversal of HIRI-related transcriptional dysregulation. Pathway enrichment analysis (Fig. 2Q and R) identified key biological processes regulated by CPs@HA. Consistent with its antioxidant phenotype, CPs@HA significantly suppressed HIRI-induced ROS-related signaling pathways, including abnormal activation of downstream targets and overexpression of oxidative stress-responsive genes. Regarding inflammatory regulation, CPs@HA downregulated pro-inflammatory pathways (NF-*κ*B, TNF, and IL-17 signaling) and concurrently upregulated anti-inflammatory and tissue repair-related gene sets, such as IL-4/IL-13 signaling and macrophage M2 polarization genes. These transcriptomic changes aligned with the observed macrophage phenotype switching and inflammatory factor expression, confirming that CPs@HA promotes the transition of the liver microenvironment from a pro-inflammatory to a reparative state. In conclusion, RNA-seq provides global evidence for the multi-target regulatory capability of CPs@HA, which inhibits ROS-related pathways and facilitates the overall transition of liver tissue from a pro-inflammatory to an anti-inflammatory state, offering systematic molecular evidence for its protective mechanism.

### 2.3 CPs@HA inhibits multiple forms of cell death in HIRI

Our transcriptomic analysis indicated that CPs@HA globally suppresses HIRI-induced oxidative stress and inflammation-related pathways. To further elucidate its role in regulating cell fate, we focused on programmed cell death. Using characteristic gene sets of distinct programmed cell death pathways (Supporting Information Table S3), we calculated the Death Potential Index *via* ssGSEA [51] (Supporting Information Table S4) and ranked the results by statistical significance (Fig. 3A). Relative to the normal group, HIRI elevated the scores of various programmed cell death pathways, with the ferroptosis potential index (FPI), cuproptosis potential index (CPI), and disulfidptosis potential index (DPI) showing the top three significant increases. In contrast, compared with the HIRI group, CPs@HA treatment downregulated multiple cell death scores, with FPI, CPI, and DPI exhibiting the most pronounced changes. These three cell death modalities have been reported to be closely associated with oxidative stress, mitochondrial dysfunction, and inflammatory amplification in HIRI pathology [52], [53], [54]. These results suggest that CPs@HA coordinately regulates multiple programmed cell death pathways linked to redox homeostasis, thereby reshaping cell fate decisions following HIRI, particularly through marked inhibition of ferroptosis, cuproptosis, and disulfidptosis. We hypothesize that its mechanism involves maintaining intracellular metal ion homeostasis, regulating mitochondrial function, and alleviating oxidative stress burden, thereby blocking the cell death cascade driven by lipid peroxidation, protein aggregation, and energy metabolism disorders. Accordingly, subsequent analyses will focus on these three programmed cell death pathways, combining functional and histological experiments to verify the association between CPs@HA's protective effects and the inhibition of these death programs.

**Fig. 3:**
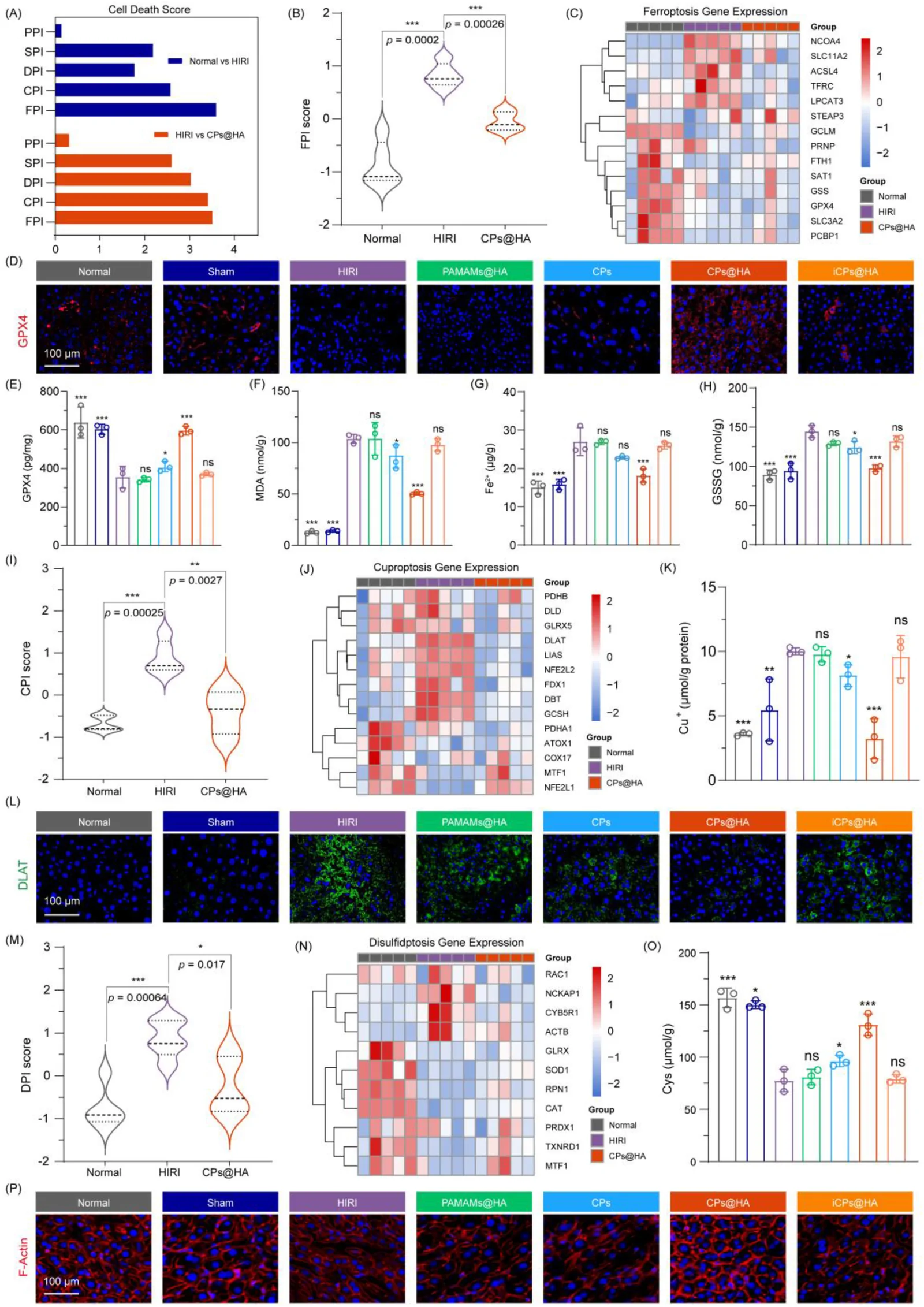
*In vivo* evaluation of the inhibitory effects of CPs@HA on multiple forms of cell death. (A) Comparison of multiple cell death scores between the HIRI and CPs@HA groups. (B) FPI in the Normal, HIRI and CPs@HA groups. (C) Heatmap of ferroptosis-related gene expression in these groups. (D, E) IF staining for GPX4 in liver tissues and quantification of the corresponding fluorescence intensity in these groups (scale bar = 100 μm). (F-H) The levels of ferroptosis-related indicators (MDA, Fe^2+^, and GSSG) in liver tissues. (I) CPI in the Normal, HIRI and CPs@HA groups. (J) Heatmap of cuproptosis-related gene expression in these groups. (K) Cu^+^ content in liver tissues. (L) IF staining for DLAT in liver tissues (scale bar = 100 μm). (M) DPI in the Normal, HIRI and CPs@HA groups. (N) Heatmap of disulfidptosis-related gene expression in these groups. (O) Cys content in liver tissues. (P) Fluorescence imaging of F-actin in liver tissues (scale bar = 100 μm). Statistical analyses were performed by comparing the HIRI group with each of the other groups (*n* = 3). *P < 0.05, **P < 0.01, ***P < 0.001 vs. indicated. ns, not significant.

Given the critical role of ferroptosis in HIRI [55], [56], we first analyzed its score changes. As shown in Fig. 3B, the FPI in the HIRI group was significantly higher than that in the Normal group, indicating that IR markedly enhanced ferroptosis susceptibility. In contrast, CPs@HA treatment substantially reduced the FPI, restoring it to near-Normal levels, demonstrating its ability to effectively lower HIRI-induced ferroptosis risk. We further analyzed the expression of ferroptosis-related genes (Fig. 3C). In the HIRI group, pro-ferroptosis genes involved in iron uptake, iron homeostasis, and lipid peroxidation susceptibility (NCOA4, SLC11A2, TFRC, ACSL4, LPCAT3, STEAP3) were upregulated, while GSH–GPX4 axis genes (GPX4, GSS, SLC3A2, GCLM) were downregulated, indicating enhanced ferroptosis susceptibility at the transcriptional level. Notably, the CPs@HA group effectively reversed the upregulation of pro-ferroptosis genes and restored GSH–GPX4 axis gene expression, shifting the overall pattern closer to that of the Normal group. Based on these transcriptomic clues, we validated key molecular indicators of ferroptosis. A core feature of ferroptosis is the abnormal accumulation of membrane lipid peroxides, closely linked to GSH depletion and impaired GPX4 function. As a key inhibitor of lipid peroxidation, GPX4 utilizes GSH to reduce harmful lipid hydroperoxides to less toxic lipid alcohols, thereby terminating the lipid peroxidation chain reaction. IF staining results (Fig. 3D and Supporting Information S33) showed that GPX4 signals were markedly reduced in the HIRI group but significantly restored in the CPs@HA group. ELISA quantification further confirmed this trend: GPX4 levels in the CPs@HA group reached 596.13 pg/mL, a 1.68-fold increase compared with the HIRI group, restoring levels comparable to those in the Normal and Sham groups (Fig. 3E). To verify whether GPX4 restoration was accompanied by alleviation of key ferroptosis events, we measured MDA, Fe^2+^, and GSSG levels. MDA reflects the accumulation of lipid peroxidation end products; Fe^2+^ can amplify lipid peroxidation through the Fenton reaction; and elevated GSSG indicates sustained oxidation of the GSH system and decreased cellular reducing capacity. As shown in Fig. 3F–H, these indicators were remarkably elevated in the HIRI group; however, after CPs@HA treatment, MDA, Fe^2+^, and GSSG levels dramatically decreased to 0.49-fold, 0.67-fold, and 0.68-fold of HIRI levels, respectively, restoring them to near-Normal and Sham levels, correlating with the GPX4 restoration trend and indicating effective intervention along the pathological axis of iron load, lipid peroxidation, and reducing capacity depletion.

Beyond ferroptosis, the transcriptomic cell death scores also indicated that cuproptosis-related programs were activated under HIRI conditions. As shown in Fig. 3I, the CPI was significantly higher in the HIRI group than in the Normal group, while CPs@HA treatment reduced it toward Normal levels, demonstrating that CPs@HA attenuates HIRI-induced cuproptosis-related transcriptional activation. We further performed clustering analysis of pro- and anti-cuproptosis gene sets using a heatmap (Fig. 3J). In the HIRI group, mitochondrial metabolism and lipoylation genes core to cuproptosis (FDX1, LIAS, DLAT, DLD, PDHA1/PDHB, DBT, GCSH) showed aberrant fluctuations. Genes related to copper homeostasis and redox regulation (ATOX1, COX17, MT1, GLRX5, NFE2L1, NFE2L2) also exhibited expression patterns inconsistent with the Normal group. After CPs@HA treatment, the overall expression pattern of these genes trended toward normalization. Based on these transcriptomic clues, we experimentally validated the cuproptosis intervention effect of CPs@HA using key molecular and biochemical indicators. Cuproptosis is driven by abnormal accumulation of reduced copper (Cu^+^), which triggers aberrant interactions of lipoylated proteins in the mitochondrial tricarboxylic acid (TCA) cycle and disrupts protein homeostasis. DLAT, a classic lipoylated protein, is a key histological indicator of cuproptosis [57], [58]. Therefore, we measured Cu^+^ content and performed DLAT IF staining (Fig. 3K and L). The results showed that Cu^+^ levels were substantially elevated in the HIRI group (up to 10.02 μmol/g protein), accompanied by stronger punctate DLAT signals, indicating abnormal DLAT oligomerization. After CPs@HA treatment, Cu^+^ content significantly decreased to 3.21 μmol/g protein (approximately 32% of the HIRI group level), and DLAT oligomerization restored to near-Normal and Sham group levels (Supporting Information Fig. S34).

We next evaluated disulfidptosis risk. As shown in Fig. 3M, the DPI showed a trend consistent with the two aforementioned cell death scores: elevated in the HIRI group but decreased after CPs@HA treatment, suggesting that CPs@HA reduces disulfidptosis-related transcriptional susceptibility. Clustering analysis of disulfidptosis-related gene sets using a heatmap (Fig. 3N) revealed that, in the HIRI group, genes involved in cytoskeleton regulation (RAC1, ACTB, NCKAP1) and redox homeostasis maintenance (TXNRD1, PRDX1, CAT, SOD1, GLRX) showed coordinated expression remodeling. After CPs@HA treatment, these aberrant expression patterns were effectively normalized, trending toward the Normal group pattern. Based on these transcriptomic clues, we validated the intervention effect of CPs@HA on disulfidptosis at metabolic and functional levels. Disulfidptosis is driven by disruption of thiol/disulfide homeostasis due to limited intracellular reducing capacity [11]. Cysteine (Cys) is a key precursor for maintaining cellular redox homeostasis, and its depletion directly increases disulfidptosis susceptibility [59]. As shown in Fig. 3O, Cys content in liver tissue was significantly reduced to 77.59 μmol/g in the HIRI group but restored to 131.01 μmol/g by CPs@HA treatment, approaching normal levels (156.68 μmol/g). In contrast, recovery in the iCPs@HA and PAMAMs@HA groups was limited, and CPs treatment showed markedly weaker effects than CPs@HA. Another key feature of disulfidptosis is disulfide stress-induced collapse and rearrangement of the actin cytoskeleton (F-actin) network. Using F-actin morphology as a histological indicator, we found that the Normal group displayed continuous, well-ordered F-actin fibers (Fig. 3P), whereas the HIRI, PAMAMs@HA, and iCPs@HA groups exhibited marked decreases in fiber continuity and network disorganization. However, after CPs@HA treatment, F-actin structure significantly improved, with fibers recovering to a continuous, well-ordered state.

Collectively, the above results consistently demonstrate the superior inhibitory effects of CPs@HA on all three cell death pathways. Mechanistically, CPs@HA suppressed ferroptosis by restoring GPX4 and reducing MDA, Fe^2+^, and GSSG levels; inhibited cuproptosis by decreasing Cu^+^ accumulation and ameliorating DLAT oligomerization; and alleviated disulfidptosis by replenishing Cys and preserving F-actin integrity.

### 2.4 Molecular mechanisms of CPs@HA in regulating ferroptosis

Having established that CPs@HA inhibits HIRI-related ferroptosis *in vivo*, we next investigated its underlying molecular mechanisms using an *in vitro* model of RSL3-induced ferroptosis in HepG2 cells. The overall workflow for the ferroptosis, cuproptosis, and disulfidptosis cell models is summarized in Supporting Information Fig. S35. RSL3, a classical ferroptosis inducer, directly inhibits GPX4, leading to rapid lipid peroxidation [60]. CCK-8 assays showed that RSL3 reduced HepG2 cell viability in a dose-dependent manner (Supporting Information Fig. S36). The concentration of 8 μg/mL reduced viability to approximately 50%, providing sufficient injury while maintaining an intervention window; therefore, it was selected for subsequent experiments. At this concentration, CPs@HA intervention significantly restored cell viability to 79.1% (Supporting Information Fig. S37) and reduced RSL3-induced PI-positive cells in live/dead staining (Supporting Information Fig. S38), confirming its potent ferroptosis-inhibitory effect.

To elucidate the molecular mechanisms, we performed RNA-seq on RSL3-treated and CPs@HA-treated cells. PCA showed clear separation between the two groups (Fig. 4A), indicating global regulation of ferroptosis-related pathways by CO. Differential expression analysis identified 2260 upregulated and 2538 downregulated genes in the CPs@HA group compared with the RSL3 group (Fig. 4B). GO and KEGG enrichment analyses revealed significant enrichment in ERK1/ERK2 signaling, cellular redox regulation, TNF/MAPK pathways, and mineral absorption (Fig. 4C), suggesting multi-target synergistic regulation of ferroptosis by CO. GSEA confirmed that CPs@HA treatment downregulated ROS (NES = 1.46, FDR < 0.25) and ferroptosis (NES = 1.7, FDR < 0.25) pathways (Fig. 4D and E). Using ssGSEA, we calculated the ferroptosis activation and inhibition scores for each sample and defined the FPI as their difference. CPs@HA treatment significantly reduced the FPI compared with the RSL3 group (*P* = 0.041, Fig. 4F). Heatmap visualization showed coordinated expression changes in pro- and anti-ferroptosis gene sets (Fig. 4G), with key ferroptosis-related genes presented in Fig. 4H.

**Fig. 4:**
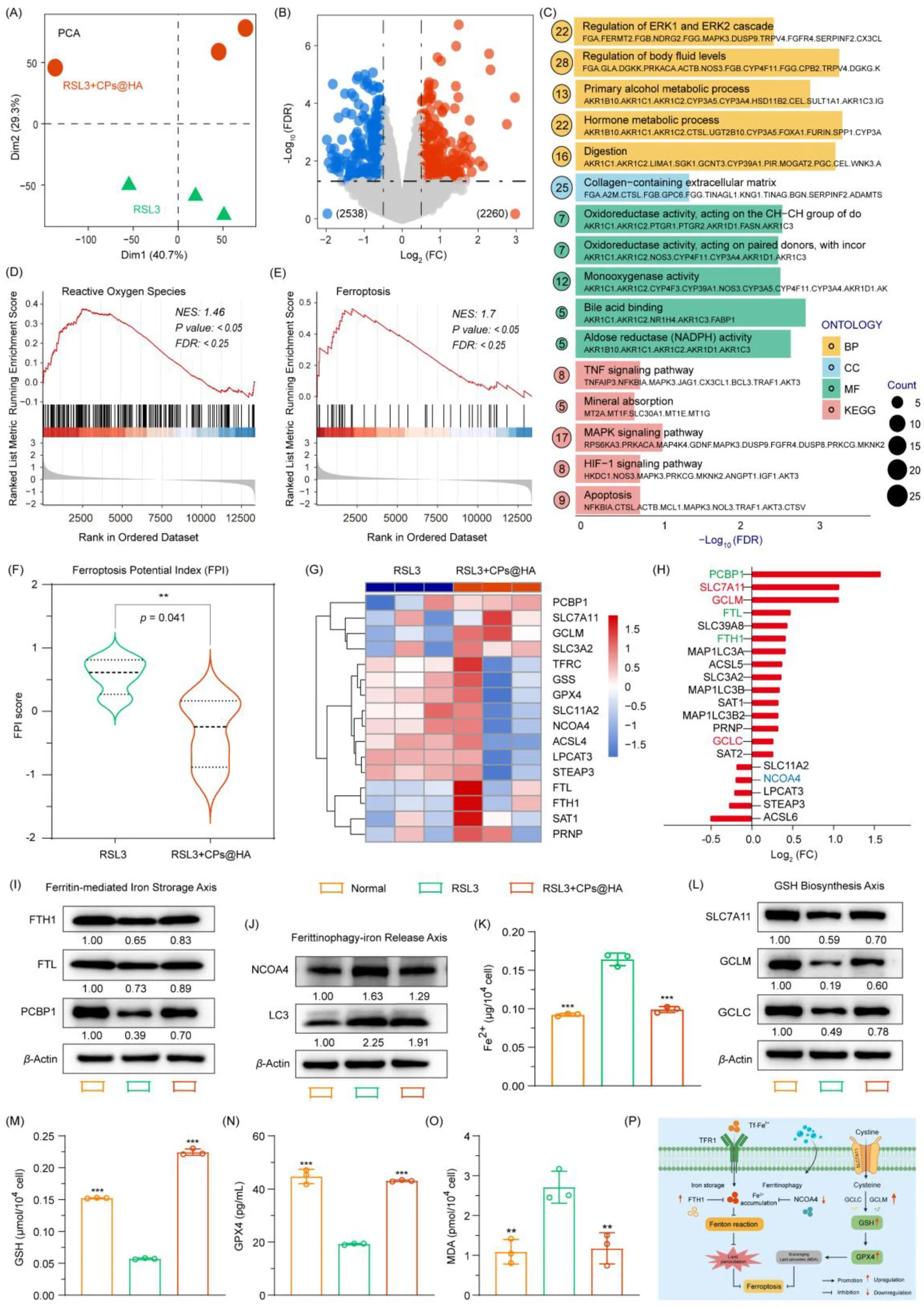
*In vitro* CPs@HA inhibits RSL3-induced ferroptosis by restoring iron homeostasis and the GSH biosynthesis axis. (A) PCA of the RSL3 and RSL3+CPs@HA groups. (B) Volcano plot of DEGs between the two groups. (C) Bar plot of GO enrichment analysis for DEGs between the two groups. (D, E) GSEA of ROS-related and ferroptosis pathways between the two groups. (F) FPI in the RSL3 and RSL3+CPs@HA groups. (G) Heatmap of ferroptosis-related gene expression in the two groups. (H) Log_2_ fold changes of representative ferroptosis-related DEGs between the two groups. (I) Expression levels of ferritin-mediated iron storage-related proteins, including FTH1, FTL, and PCBP1. (J) Expression levels of ferritinophagy-iron release axis-related proteins, including NCOA4 and LC3. (K) Intracellular Fe^2+^ content. (L) Expression levels of GSH biosynthesis axis-related proteins, including SLC7A11, GCLM and GCLC. (M − O) Intracellular levels of GSH, GPX4 and MDA. (P) Schematic illustration of the mechanism by which CPs@HA inhibits ferroptosis (created with BioRender.com). Statistical analyses were performed to compare the RSL3 group with the Normal group and with the RSL3+CPs@HA group, respectively (*n* = 3). *P < 0.05, **P < 0.01, ***P < 0.001 *vs*. indicated. ns, not significant.

Ferroptosis is primarily driven by excessive iron-dependent lipid peroxidation and is closely associated with intracellular iron metabolic homeostasis and antioxidant defense capacity. Under ferroptotic stress, the expression of iron-storage-related proteins such as FTH1 is impaired, weakening ferritin-mediated sequestration of labile iron. Meanwhile, NCOA4-mediated ferritinophagy is enhanced, promoting ferritin degradation and Fe^2+^ release, which increases intracellular labile Fe^2+^ levels. Excess labile Fe^2+^ further drives the Fenton reaction, inducing ROS generation and lipid peroxidation. At the same time, suppression of the SLC7A11–GSH–GPX4 antioxidant axis leads to insufficient GSH synthesis, reduced GPX4 activity, and impaired lipid peroxide clearance, ultimately triggering ferroptosis.

Based on this background and the above transcriptomic results, we focused on three key processes that may contribute to CPs@HA-mediated ferroptosis inhibition: iron storage, ferritinophagy-mediated iron release, and the SLC7A11–GSH–GPX4 antioxidant axis. 1) Iron storage and transport axis. Ferritin light chain (FTL), heavy chain (FTH1), and the iron chaperone PCBP1 are core components for buffering and storing intracellular labile iron. FTH1 and FTL sequester labile iron within the ferritin complex, while PCBP1 facilitates iron delivery and participates in ferritin loading [61], [62]. Western blot (WB) analysis showed that RSL3 decreased FTH1, FTL, and PCBP1 expression to 65%, 73%, and 39%, respectively, compared with the normal group (Fig. 4I), indicating impaired iron storage capacity and increased labile iron available for oxidative reactions. CPs@HA intervention restored these proteins to 83%, 89%, and 70% of normal levels, respectively, reversing the RSL3-induced impairment. 2) Ferritinophagy-mediated iron release axis. NCOA4-mediated ferritinophagy promotes ferritin degradation and labile iron release [63]. LC3, an autophagosome membrane marker, accumulates upon autophagosome formation, reflecting ferritinophagy activation [64]. In the RSL3 group, NCOA4 and LC3 expression were upregulated (1.63-fold and 2.25-fold *vs.* normal group) (Fig. 4J), indicating ferritinophagy activation and enhanced iron release. CPs@HA intervention reduced NCOA4 expression and LC3 activation to 1.29-fold and 1.91-fold of normal levels, respectively, thereby partially reducing ferritin degradation. Consistent with these protein changes, intracellular Fe^2+^ levels were elevated in the RSL3 group but restored to near-normal levels after CPs@HA treatment (Fig. 4K). Flow cytometry further confirmed that RSL3 treatment markedly increased intracellular ROS levels (78.3% ROS-positive cells), which was largely reversed by CPs@HA intervention (12.0% *vs.* normal 5.09%) (Supporting Information Fig. S39), consistent with excess Fe^2+^ driving ROS production *via*

the Fenton reaction. 3) GSH biosynthesis axis. GSH is an essential cofactor for GPX4 in scavenging lipid peroxides. Its synthesis depends on the cystine transporter SLC7A11 and the rate-limiting enzymes GCLM and GCLC [65], [66]. RSL3 treatment downregulated SLC7A11, GCLM, and GCLC expression to 59%, 19%, and 49% of normal levels, respectively (Fig. 4L), leading to impaired GSH synthesis (to 40% of normal levels, Fig. 4M). Consequently, GPX4 activity was reduced to 43% of normal levels (Fig. 4N), followed by increased GSSG accumulation (to 1.50-fold of normal levels, Supporting Information Fig. S40A) and decreased GSH/GSSG ratio (to 0.25-fold of normal levels, Fig. S40B), resulting in increased intracellular oxidative load. Notably, CPs@HA intervention restored SLC7A11, GCLM, and GCLC expression to 70%, 60%, and 78% of normal levels, respectively; improved GSH synthesis to 147% of normal levels; restored GPX4 activity to 96.24% of normal levels; decreased GSSG accumulation to near-normal levels; and increased the GSH/GSSG ratio to 1.58-fold of normal levels. These results indicate that CPs@HA effectively reestablished redox homeostasis, as evidenced by reduced labile Fe^2+^ levels and restored GSH synthesis. The reduction in labile Fe^2+^ levels and the restoration of GSH levels jointly contributed to the effective inhibition of cellular lipid peroxidation. To directly assess whether this leads to inhibition of lipid peroxidation, we measured MDA, a terminal product of this process. MDA content was elevated to 2.71 pmol/10^4^ cells in the RSL3 group but dramatically decreased to 1.17 pmol/10^4^ cells after CPs@HA intervention, approaching the normal group level (1.09 pmol/10^4^ cells) (Fig. 4O).

In summary, by releasing CO, CPs@HA systematically inhibits RSL3-induced ferroptosis through three key axes: iron storage, ferritinophagy, and GSH synthesis, as evidenced by restored iron-handling proteins, reduced ferritinophagy-mediated Fe^2+^ release (thereby lowering labile iron), and recovered GSH-GPX4 antioxidant activity, ultimately attenuating lipid peroxidation. These findings provide multi-level molecular evidence for its potential therapeutic role in ferroptosis-related diseases (Fig. 4P).

### 2.5 Molecular mechanisms of CPs@HA in regulating cuproptosis

Having validated the anti-ferroptosis effect of CPs@HA, we next investigated its regulatory mechanisms in cuproptosis using an *in vitro* model of Elesclomol (ES) and CuCl_2_ co-induced (ES/CuCl_2_) cuproptosis in HepG2 cells [67], [68]. To avoid excessive cytotoxicity that might interfere with combined effect evaluation, we first screened ES and CuCl_2_ individually to identify concentrations that are minimally toxic alone but produce stable damage when combined. Based on single-agent screening results (Supporting Information Fig. S41 and S42), we further identified the optimal modeling conditions through combination matrix experiments. The optimal combination of 100 nmol/L ES and 1 μmol/L CuCl_2_ reduced cell viability to approximately 50% (Supporting Information Fig. S43), providing sufficient injury while retaining an adequate therapeutic window. At this concentration, CPs@HA restored cell viability from 52.2% to 81.2% (Supporting Information Fig. S44), and live/dead staining further confirmed reduced cell death (Supporting Information Fig. S45), demonstrating its potent protective effect against cuproptosis.

To elucidate the molecular mechanisms, we performed RNA-seq on ES/CuCl_2_-treated and ES/CuCl_2_+CPs@HA-treated cells. PCA showed clear separation between the two groups (Fig. 5A), indicating global regulation of cuproptosis-related pathways by CO. Differential expression analysis identified 1046 upregulated and 845 downregulated genes in the CPs@HA group (Fig. 5B). GO and KEGG enrichment analyses revealed significant enrichment in wound healing, lipid homeostasis, and complement/coagulation cascades (Fig. 5C), suggesting multi-target synergistic regulation. GSEA confirmed that ES/CuCl_2_+CPs@HA treatment downregulated oxidative phosphorylation and TCA cycle pathways (NES < −1, FDR < 0.25; Fig. 5D and E), indicating alleviation of copper-driven mitochondrial metabolic stress. Given that cuproptosis results from aberrant copper interactions with lipoylated TCA proteins leading to mitochondrial aggregation, this metabolic recovery supports a CO-mediated protective effect on mitochondria.

**Fig. 5:**
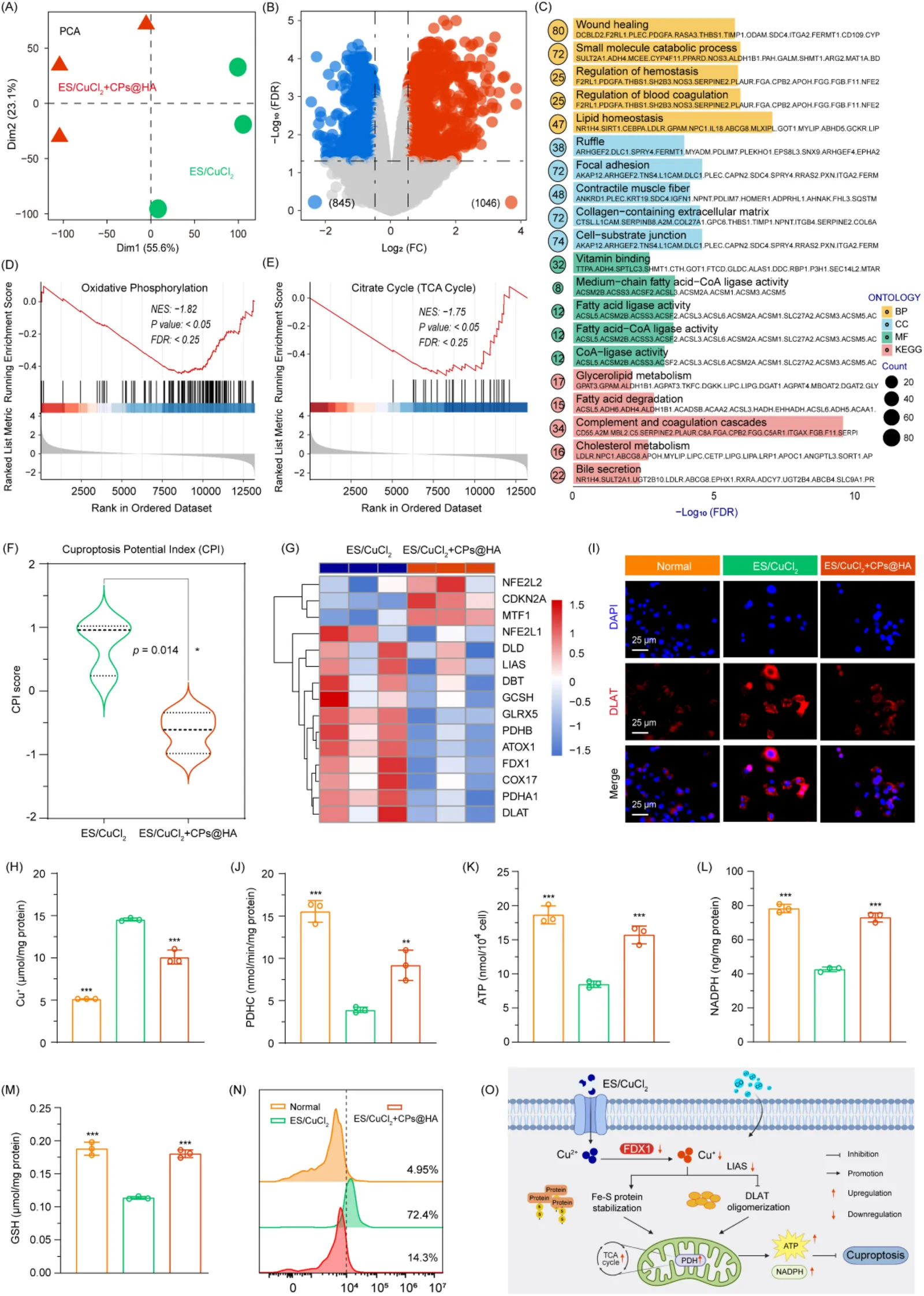
*In vitro* CPs@HA alleviates ES/CuCl_2_-induced cuproptosis by reducing Cu^+^ accumulation and restoring DLAT homeostasis. (A) PCA of the ES/CuCl_2_ and ES/CuCl_2_+CPs@HA groups. (B) Volcano plot of DEGs between the two groups. (C) Bar plot of GO enrichment analysis for DEGs between the two groups. (D, E) GSEA of oxidative phosphorylation and TCA cycle pathways between the two groups. (F) CPI in the ES/CuCl_2_ and ES/CuCl_2_+CPs@HA groups. (G) Heatmap of cuproptosis-related gene expression in the two groups. (H) Intracellular Cu^+^ content. (I) IF staining for intracellular DLAT (scale bar = 25 μm). (J-N) Intracellular PDHC activity, ATP and NADPH levels, GSH content, and ROS levels. (O) Schematic illustration of the mechanism by which CPs@HA inhibits cuproptosis (created with BioRender.com). Statistical analyses were performed by comparing the ES/CuCl_2_ group with each of the other groups (*n* = 3). *P < 0.05, **P < 0.01, ***P < 0.001 vs. indicated. ns, not significant.

To quantitatively evaluate cuproptosis propensity, we used ssGSEA to calculate cuproptosis activation and inhibition scores for each sample and defined the CPI as their difference. The CPI of the ES/CuCl_2_+CPs@HA group was significantly lower than that of the ES/CuCl_2_ group (*P* < 0.05, Fig. 5F), indicating that CO attenuates cuproptosis susceptibility and enhances cellular tolerance to copper overload. Heatmap analysis (Fig. 5G) revealed upregulation of antioxidant-related genes (NFE2L2/NRF2, MTF1, CDKN2A) and downregulation of energy metabolism and copper reduction genes (FDX1, LIAS, DLD, PDHB, PDHA1, DLAT).

Based on these transcriptomic clues, we next validated the key molecular events of cuproptosis. Abnormal accumulation of reduced Cu^+^ is a central driver of this process. FDX1, a key reductase, promotes the reduction of Cu^2+^ to Cu. Meanwhile, LIAS catalyzes the lipoylation of DLAT to generate lipoylated DLAT [57], [69]. Under copper overload, Cu^+^ aberrantly binds to lipoylated DLAT, inducing its oligomerization/aggregation, which disrupts mitochondrial homeostasis and consequently impairs PDH complex function as well as TCA cycle activity. In parallel, Cu^+^ destabilizes Fe-S cluster proteins, further aggravating mitochondrial metabolic dysfunction. Collectively, these changes suppress the TCA cycle, reduce ATP production, decrease NADPH levels, impair cellular energy supply and redox capacity, and ultimately promote cuproptosis [70].

Transcriptomic analysis revealed that ES/CuCl_2_+CPs@HA treatment downregulated FDX1 and LIAS expression (Fig. 5G). Their downregulation suggests reduced Cu^+^ accumulation and impaired lipoylation under ES/CuCl_2_+CPs@HA treatment. Accordingly, it is predicted that ES/CuCl_2_+CPs@HA could lower intracellular Cu^+^ levels and mitigate DLAT oligomerization. We therefore measured intracellular Cu^+^ content and DLAT oligomerization. As shown in Fig. 5H, Cu^+^ content was remarkably lower in the ES/CuCl_2_+CPs@HA group than in the ES/CuCl_2_ group (10.08 *vs.* 14.53 μmol/mg prot). IF staining further revealed significantly enhanced DLAT signals with an abnormal punctate distribution in ES/CuCl_2_-treated cells, indicating impaired DLAT homeostasis (Fig. 5I and Supporting Information S46). Notably, after ES/CuCl_2_+CPs@HA treatment, DLAT distribution resembled that of the control group. These results demonstrate that CPs@HA regulates copper homeostasis to inhibit cuproptosis.

Having established that CPs@HA reduces Cu^+^ accumulation and restores DLAT homeostasis, we next investigated how these protective effects impact the downstream mitochondrial metabolic network. In the absence of intervention, Cu^+^ overload and DLAT oligomerization are known to propagate to mitochondria, manifesting as energy deficit and oxidative stress [71]. We measured pyruvate dehydrogenase (PDH) activity and ATP levels as readouts of mitochondrial status. PDH activity reflects both pyruvate entry into the TCA cycle and PDH complex function, while ATP levels indicate mitochondrial energy production [72], [73]. Both were significantly decreased after ES/CuCl_2_ treatment (Fig. 5J and K): PDH activity fell from 15.58 to 3.93 nmol/min/mg prot, and ATP levels dropped from 19.72 to 8.97 nmol/10^4^ cells, indicating impaired energy supply. CPs@HA intervention restored PDH activity to 9.23 nmol/min/mg prot (approximately 2.35-fold of the ES/CuCl_2_ group level) and elevated ATP levels to 16.61 nmol/10^4^ cells (approximately 1.85-fold of the ES/CuCl_2_ group level), supporting its protective effect.

Given that mitochondrial energy production was restored by CPs@HA (as shown by PDH activity and ATP levels), we next examined whether this metabolic rescue also restored cellular redox homeostasis. NADPH, a key indicator of reducing capacity, provides reducing equivalents for GSH regeneration; its depletion disrupts redox homeostasis [74]. ES/CuCl_2_ treatment reduced NADPH from 78.29 to 42.58 ng/mg prot (Fig. 5L), accompanied by decreased GSH (to 0.11 μmol/mg prot, Fig. 5M) and increased GSSG (to 1.48-fold of normal levels, Supporting Information Fig. S47), indicating a shift toward an oxidative state. CPs@HA intervention replenished NADPH to 73.03 ng/mg prot, increased GSH to 0.18 μmol/mg prot, and reduced GSSG to 1.02-fold of normal levels (Fig. 5M and Supporting Information S47). Consistent with these biochemical changes, ROS-positive cells surged to 72.4% following ES/CuCl_2_ treatment but were reduced to 14.3% by ES/CuCl_2_+CPs@HA treatment, approaching the normal level (4.95%, Fig. 5N).

In summary, CPs@HA systematically inhibits ES/CuCl_2_-induced cuproptosis through multiple mechanisms: regulating copper homeostasis, protecting mitochondrial metabolism, and restoring redox homeostasis. These findings provide multi-level molecular evidence for its potential therapeutic role in cuproptosis-related diseases (Fig. 5O).

### 2.6 Molecular mechanisms of CPs@HA in regulating disulfidptosis

Having investigated the mechanisms of ferroptosis and cuproptosis, we next studied how CPs@HA regulates disulfidptosis using a glucose starvation-induced model in HepG2 cells [75], [76]. CCK-8 assays showed that cell viability decreased progressively with prolonged starvation, reaching 50% at 36 h, which was therefore selected as the standard treatment time (Supporting Information Fig. S48). Under this condition, CPs@HA intervention restored cell viability to 76.13% (Supporting Information Fig. S49), indicating that CO alleviates cell damage under energy stress and thus provides a basis for subsequent mechanistic validation.

To elucidate the molecular mechanisms of CO, we performed RNA-seq on glucose-deprived (Glu(−)) and CPs@HA-treated cells. PCA revealed clear separation between the two groups (Fig. 6A), indicating that CO globally regulates disulfidptosis-related pathways. Differential expression analysis identified 2389 upregulated and 4178 downregulated genes in the CPs@HA group (Fig. 6B). GO and KEGG enrichment analyses showed significant enrichment in DNA replication, chromosome condensation, oxidoreductase activity, and actin cytoskeleton regulation (Fig. 6C), suggesting multi-target synergistic regulation of disulfidptosis by CO. GSEA further confirmed that CPs@HA treatment significantly upregulated glycolysis and cell cycle pathways (NES > 1, FDR < 0.25; Fig. 6D and E), indicating that CO enables cells to overcome energy metabolism challenges induced by glucose deprivation, thereby maintaining energy supply and proliferative capacity. During early starvation, cells upregulate glycolysis and cell cycle genes to sustain energy production and proliferation; however, prolonged glucose deprivation and metabolic burden lead to energy insufficiency, which in turn triggers cell cycle inhibition and senescence, a protective response that arrests the cell cycle under adverse conditions.

**Fig. 6:**
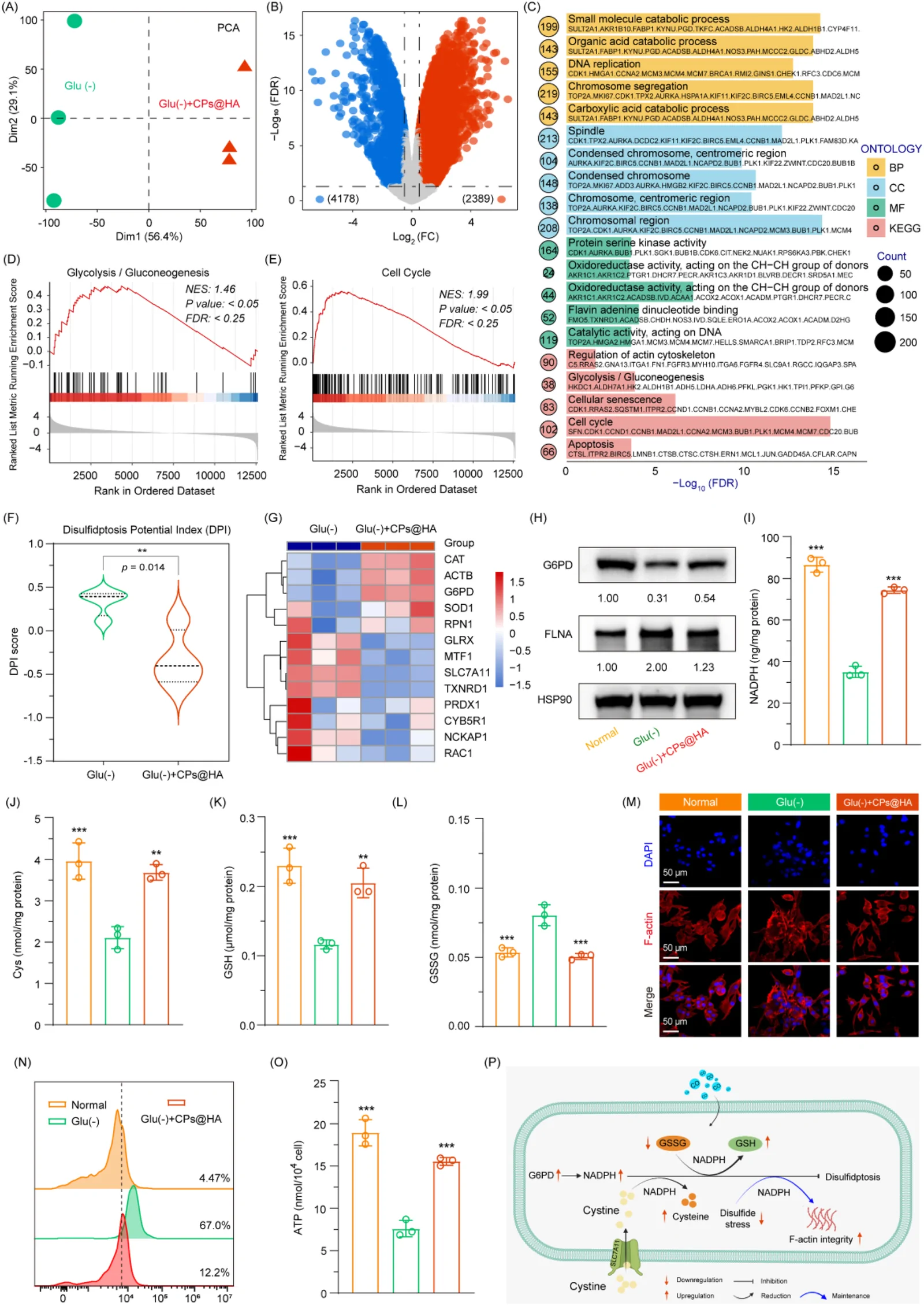
*In vitro* CPs@HA inhibits Glu(−)-induced disulfidptosis by restoring G6PD/NADPH to replenish Cys and GSH levels and alleviating F-actin disruption caused by disulfide stress. (A) PCA of the Glu(−) and Glu(−) + CPs@HA groups. (B) Volcano plot of DEGs between the two groups. (C) Bar plot of GO enrichment analysis for DEGs between the two groups. (D, E) GSEA of glycolysis/gluconeogenesis and cell cycle pathways in the two groups. (F) DPI in the Glu(−) and Glu(−) + CPs@HA groups. (G) Heatmap of disulfidptosis-related gene expression in the two groups. (H) Expression levels of the disulfidptosis-related proteins G6PD and FLNA. (I-L) Intracellular levels of NADPH, Cys, GSH, and GSSG. (M) Fluorescence imaging of F-actin (scale bar = 50 μm). (A) Flow cytometric analysis of intracellular ROS levels. (O) Intracellular levels of ATP. (P) Schematic illustration of the mechanism by which CPs@HA inhibits disulfidptosis (created with BioRender.com). Statistical analyses were performed by comparing the Glu(−) group with each of the other groups (*n* = 3). *P < 0.05, **P < 0.01, ***P < 0.001 vs. indicated. ns, not significant.

Following the same analytical steps used for ferroptosis and cuproptosis, we quantitatively evaluated the cellular propensity for disulfidptosis. Using ssGSEA, we calculated the disulfidptosis activation and inhibition scores for each sample and defined the DPI as their difference. The DPI of the CPs@HA group was significantly lower than that of the Glu(−) group (*P* < 0.05; Fig. 6F), indicating that CO attenuates disulfidptosis susceptibility and enhances cellular tolerance. Heatmap analysis (Fig. 6G) revealed upregulation of antioxidant-related genes (CAT, SOD1, G6PD) and downregulation of the amino acid transport-related gene SLC7A11.

Based on these transcriptomic clues, we next validated the key molecular events of disulfidptosis. Given that NADPH depletion is the core driver of disulfidptosis under glucose deprivation, we focused on verifying NADPH metabolism and its downstream consequences. Mechanistically, glucose deprivation impairs NADPH production *via* the pentose phosphate pathway (PPP), while SLC7A11-mediated cystine uptake and its reduction to cysteine continuously consume NADPH, further exacerbating its depletion [11], [77]. When reducing reserves become insufficient, intracellular disulfide pressure increases, leading to aberrant protein disulfide crosslinking and rapid cell death [11]. Transcriptomic analysis (Fig. 6G) revealed upregulation of G6PD, a key enzyme in the PPP that generates NADPH, as well as downregulation of SLC7A11, which mediates NADPH-consuming cystine uptake. These transcriptomic changes suggest that CPs@HA may restore NADPH levels under glucose deprivation.

To further verify the upregulation of G6PD by CPs@HA and determine whether it alleviates NADPH depletion under glucose deprivation, we next examined G6PD expression and NADPH levels. WB analysis showed that G6PD expression was markedly downregulated under glucose deprivation (Fig. 6H), consistent with the observed decrease in NADPH levels. Specifically, glucose deprivation reduced NADPH from 86.70 to 36.08 ng/mg prot (Fig. 6I). NADPH depletion impaired cystine reduction, lowering free cysteine content from 3.96 to 2.11 nmol/mg protein (Fig. 6J), which further compromised protein thiol maintenance and promoted aberrant disulfide formation [78]. In addition, NADPH depletion caused GSH/GSSG redox imbalance, as evidenced by decreased GSH (from 0.23 to 0.12 μmol/mg prot) and increased GSSG (from 0.05 to 0.08 nmol/10^4^ cells) (Fig. 6K and L). This indicates impaired GSH cycling, reduced thiol reducing capacity, and amplified disulfide stress. Notably, CPs@HA treatment restored NADPH levels to 74.44 ng/mg prot, improved cysteine levels to 3.69 nmol/mg prot, and reversed the GSH/GSSG redox imbalance (increasing GSH to 0.21 μmol/mg prot and decreasing GSSG to 0.05 nmol/10^4^ cells). These results suggest that CO alleviates disulfidptosis by restoring reducing reserves, as evidenced by the replenishment of cysteine and the rebalancing of GSH/GSSG redox status, thereby reducing disulfide stress.

Having established that CPs@HA restores reducing reserves and thiol homeostasis, we next examined whether it also protects the actin cytoskeleton, another hallmark of disulfidptosis. Disulfide stress is known to cause collapse and rearrangement of the actin cytoskeleton. WB analysis (Fig. 6H) revealed that under glucose deprivation, FLNA, a key scaffold molecule of the actin cytoskeleton, was significantly upregulated, suggesting cytoskeletal remodeling in response to disulfide stress [79]. Consistent with these molecular changes, F-actin staining showed stress fiber collapse-like rearrangement (Fig. 6M), mirroring the cytoskeletal disintegration phenotype characteristic of disulfidptosis [11]. CPs@HA treatment effectively reversed these abnormalities, restoring FLNA expression and preserving continuous F-actin filamentous structures that approached normal control levels.

Based on these metabolic and cytoskeletal findings, we further evaluated oxidative stress and energy metabolism as functional readouts. Flow cytometry showed that glucose deprivation increased the proportion of ROS-positive cells to 67.0%, while CPs@HA reduced this proportion to 12.2%, slightly above the normal group level of 4.47% (Fig. 6N), indicating effective alleviation of oxidative stress. Meanwhile, ATP levels decreased to 7.59 nmol/10^4^ cells under glucose deprivation and recovered to 15.55 nmol/10^4^ cells after CPs@HA treatment, approaching the normal group level of 18.94 nmol/10^4^ cells (Fig. 6O), demonstrating improved energy metabolism.

In summary, CPs@HA systematically inhibits glucose starvation-induced disulfidptosis by restoring reducing capacity, maintaining thiol homeostasis, and protecting the cytoskeleton, providing molecular evidence for its therapeutic potential in disulfidptosis-related diseases (Fig. 6P).

### 2.7 CPs@HA-mediated mitochondrial homeostasis restoration

Based on the aforementioned *in vivo* and *in vitro* results, CPs@HA significantly alleviates HIRI-induced liver damage by concurrently suppressing ferroptosis, cuproptosis, and disulfidptosis. This protective effect is evident both in the restoration of pathway-specific markers (GPX4 restoration, reduced DLAT oligomerization, and F-actin cytoskeleton reconstruction) and in the synergistic improvement of shared injury indicators, including alleviation of Fe^2+^ and Cu ^+^ overload, reduction in ROS levels, mitigation of NADPH depletion, normalization of the GSH/GSSG redox imbalance, and recovery of ATP levels. This multi-target, cross-pathway synergistic regulation suggests that the protective mechanism of CPs@HA does not simply act on the terminal execution steps of these cell death pathways individually, but rather intervenes upstream in the key pathogenic foundation upon which these pathways commonly depend.

Mitochondria serve as the central hub for cellular energy metabolism, redox balance, and ion homeostasis, and thus play a critical role in HIRI pathogenesis. Numerous studies have shown that mitochondrial dysfunction is highly coupled with HIRI progression: the early reperfusion phase is typically marked by mitochondrial Ca^2+^ overload, decreased ΔΨm, and impaired respiratory chain complex activity, which trigger a burst of mtROS and thereby reduce ATP synthesis. Subsequently, opening of the mPTP, loss of membrane integrity, and release of pro-apoptotic signals further amplify oxidative stress and metabolic dysregulation [80], [81], [82], [83], [84]. Importantly, these mitochondrial damage phenotypes, including metal ion homeostasis imbalance, redox dysregulation, and energy failure, closely mirror the abnormal metal ion overload, NADPH depletion, and ATP decline observed in our study. This suggests that mitochondrial dysfunction underlies the parallel activation of ferroptosis, cuproptosis, and disulfidptosis. Based on this rationale, we further investigated whether CPs@HA protects mitochondrial structure and function, aiming to elucidate how CO, by restoring mitochondrial homeostasis and blocking the injury amplification cascade, synergistically inhibits multiple cell death pathways. This establishes an evidence chain linking "mitochondrial protection" to "multi-pathway cell death inhibition".

To gain mechanistic insights, we performed RNA-seq on liver tissues from the Normal, HIRI, and CPs@HA groups. GSEA revealed that, compared with the HIRI group, the CPs@HA group exhibited significant downregulation of the gene sets "Response to hypoxia" and "Response to oxidative stress" (NES > 1, *P* < 0.05, FDR < 0.25; Fig. 7A and B). This suggests that CO released by CPs@HA attenuates stress-related transcriptional programs driven by oxygen imbalance and ROS burst during reperfusion, consistent with its amelioration of the HIRI microenvironment and oxidative damage. Further analysis showed that CPs@HA treatment significantly upregulated multiple mitochondria-associated genes, including *SIRT1* and *PPARGC1A* (encoding PGC-1*α*), which are key regulators of mitochondrial biogenesis and antioxidant defense, as well as the antioxidant enzymes *HMOX2*, *SOD1*, and *CAT* (Fig. 7C). Activation of these genes enhances mitochondrial fitness and quality control [85], [86], thereby mitigating ion homeostasis disruption and the exacerbated oxidative stress caused by energy supply-demand imbalance during reperfusion. To further explore the transcriptomic clues related to ion homeostasis, we performed enrichment analysis of Ca^2+^ homeostasis-related pathways. Multiple Ca^2+^-related GO terms were significantly enriched in the HIRI group, including Ca^2+^ homeostasis/intracellular Ca^2+^ homeostasis, regulation of cytosolic Ca^2+^ concentration, and Ca^2+^ transport/Ca^2+^ transmembrane import into the cytosol (Fig. 7D). This indicates that HIRI simultaneously affects Ca^2+^ transmembrane entry, cytosolic concentration changes, and transport processes, suggesting dysregulation of the Ca^2+^ homeostatic system under reperfusion stress. In contrast, in the CPs@HA group, the distribution of these terms shifted toward regulation and homeostasis maintenance (Fig. 7E), including Ca^2+^ homeostasis/intracellular Ca^2+^ homeostasis, positive regulation of Ca^2+^ import, positive/negative regulation of cytosolic Ca^2+^ concentration, and positive regulation of Ca^2+^ import. This remodeling indicates that after CPs@HA intervention, Ca^2+^-related pathways shift from those associated with elevated cytosolic Ca^2+^ load (in HIRI) toward those involved in homeostasis maintenance and concentration regulation.

**Fig. 7:**
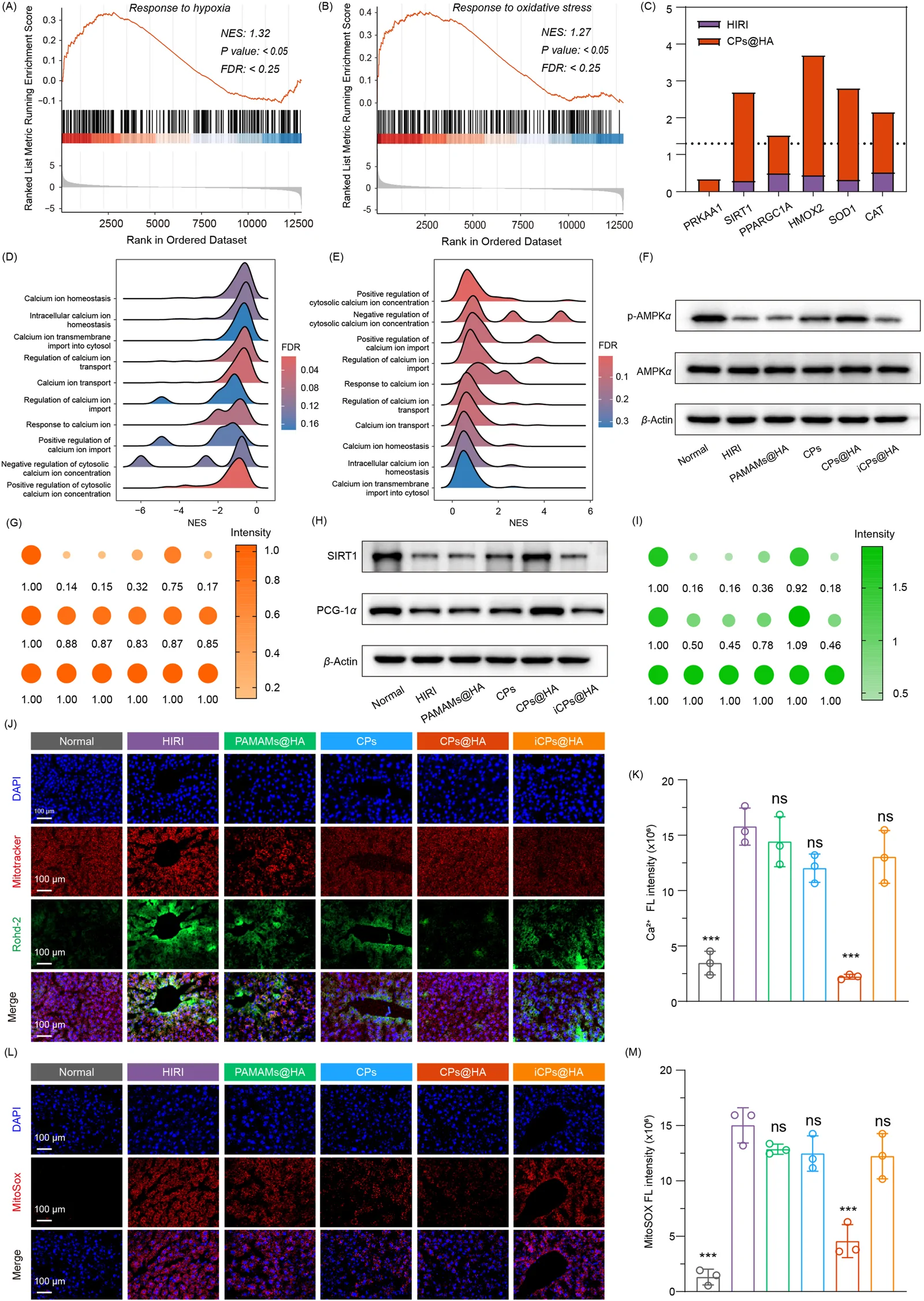
*In vivo* CPs@HA restores mitochondrial homeostasis by activating the AMPK–SIRT1–PGC-1α axis to alleviate Ca^2+^ overload and mtROS burst during HIRI. (A, B) GSEA of the response to hypoxia and response to oxidative stress pathways in the HIRI and CPs@HA groups. (C) Changes in the expression of key mitochondrial genes in the two groups. (D) Ridge plot of GO enrichment analysis related to calcium homeostasis in the Normal and HIRI groups. (E) Ridge plot of GO enrichment analysis related to calcium homeostasis in the HIRI and CPs@HA groups. (F, G) Protein expression levels of p-AMPKα and AMPKα in liver tissues, and the corresponding densitometric quantification of the bands. (H, I) Protein expression levels of SIRT1 and PGC-1α in liver tissues, and the corresponding densitometric quantification of the bands. (J, K) Fluorescence imaging of mitochondrial Ca^2+^ in liver tissues and the corresponding quantitative analysis of fluorescence intensity (scale bar = 100 μm). (L, M) Fluorescence imaging of mtROS in liver tissues and the corresponding quantitative analysis of fluorescence intensity (scale bar = 100 μm). Statistical analyses were performed by comparing the HIRI group with each of the other groups (*n* = 3). *P < 0.05, **P < 0.01, ***P < 0.001 vs. indicated. ns, not significant.

These transcriptomic changes, including upregulation of SIRT1/PGC-1*α* and remodeling of Ca^2+^-related pathways, prompted us to investigate the upstream signaling axis that coordinates mitochondrial biogenesis, Ca^2+^ homeostasis, and redox balance. The AMPK–SIRT1–PGC-1*α* axis is a central regulator of mitochondrial functional recovery. After CPs@HA treatment, this axis is activated and subsequently promotes mitochondrial functional recovery. As an energy sensor, AMPK facilitates metabolic recovery and initiates mitochondrial protective responses. SIRT1 is a deacetylase closely associated with cellular energy and redox status, and it regulates mitochondrial metabolism and antioxidant responses. PGC-1*α* is a key regulator of mitochondrial biogenesis and function; its activation contributes to improved mitochondrial respiration and energy production. Collectively, these findings suggest that CO, by activating the AMPK–SIRT1–PGC-1*α* axis, enhances mitochondrial energy metabolism and antioxidant capacity. In line with this, previous studies have shown that SIRT1 is involved in regulating mitochondrial Ca^2+^ uptake, and its activation may help alleviate mitochondrial Ca^2+^ overload, thereby suppressing excessive mtROS generation [87], [88]. Moreover, CO can directly regulate Ca^2+^ homeostasis. In cardiomyocyte models, CO directly inhibits L-type Ca^2+^ channel currents and reduces Ca^2+^ influx. In airway epithelial cells, CO regulates intracellular Ca^2+^ signaling through P2Y2/P4 and PLC-IP_3_ pathways and suppresses both Ca^2+^ store release and extracellular Ca^2+^ influx upon UTP stimulation [89], [90]. Based on this rationale, we hypothesized that CPs@HA, by releasing CO, activates the AMPK–SIRT1–PGC-1*α* axis and restores Ca^2+^ homeostasis, collaboratively suppressing mtROS generation and ultimately restoring mitochondrial homeostasis.

To validate these transcriptomic clues, we examined key molecules of the AMPK–SIRT1–PGC-1*α* axis by WB. *p*-AMPK*α* levels in the HIRI group were decreased to 14% of normal levels, while total AMPK*α* remained unchanged (Fig. 7F and G), indicating that HIRI primarily inhibits AMPK phosphorylation and activation. Concomitant with upstream *p***-**AMPK inhibition, downstream SIRT1 and PGC-1*α* expression was also downregulated, to 16% and 50% of normal levels, respectively (Fig. 7H and I). CPs@HA intervention restored *p*-AMPK*α* to 75% of normal levels and upregulated SIRT1 and PGC-1*α* to 92% and 109% of normal levels, consistent with changes of the entire signaling axis. Together, these results indicate that changes in this axis are associated with mitochondrial homeostasis and functional recovery.

To validate the predicted restoration of Ca^2+^ homeostasis, we detected mitochondrial Ca^2+^ load using Rhod-2 staining. Rhod-2 fluorescence was significantly enhanced in the HIRI group and co-localized with the mitochondrial probe (MitoTracker). Line scan intensity curves of Rhod-2 and MitoTracker exhibited adjacent peaks and synchronous changes at multiple positions (Pearson's r = 0.7431), indicating enrichment of Ca^2+^ signals in MitoTracker-labeled regions (Fig. 7J and Supporting Information Fig. S50). The relative Ca^2+^ fluorescence intensity was markedly increased in the HIRI group, whereas CPs@HA treatment reduced it from approximately 0.14-fold of the HIRI level, indicating effective alleviation of HIRI-induced abnormal mitochondrial Ca^2+^ overload (Fig. 7K). Together, these results demonstrate that CPs@HA improves Ca^2+^ homeostasis, thereby blocking the mitochondrial damage cascade driven by Ca^2+^ imbalance during early reperfusion. Given the reciprocal relationship between Ca^2+^ overload and oxidative stress, we next considered the underlying mechanisms.

A key mechanism involves a vicious injury cycle in which Ca^2+^ imbalance disrupts mitochondrial metabolism and electron transport chain function, increasing electron leakage and promoting mtROS generation, while also inducing mPTP opening that further amplifies ROS load [91], [92]. Based on this, we examined the effect of CPs@HA on mtROS levels. MitoSOX staining showed that mtROS fluorescence intensity was significantly enhanced in the HIRI group, increasing by 38.15% compared with the Normal group, indicating a burst of mitochondrial oxidative stress induced by reperfusion (Fig. 7L). In contrast, mtROS fluorescence intensity was significantly decreased after CPs@HA intervention, approaching normal levels (Fig. 7M). Together, these results demonstrate that CPs@HA effectively blocks the synergistic amplification loop between Ca^2+^ and mtROS, exhibiting superior efficacy in inhibiting mitochondrial oxidative stress.

Collectively, these *in vitro* findings validate our hypothesis that CPs@HA, by releasing CO, activates the AMPK–SIRT1–PGC-1*α* axis and restores Ca^2+^ homeostasis, thereby collaboratively suppressing mtROS generation and ultimately preserving mitochondrial homeostasis. Having established these molecular and functional recoveries, we next sought to determine whether they translate into the restoration of mitochondrial integrity and comprehensive function, including membrane potential, ultrastructure, respiratory chain capacity, and energy/redox balance.

To address this, we first evaluated ΔΨm by JC-1 staining and observed mitochondrial ultrastructure by TEM, given that Ca^2+^ overload and mtROS burst can synergistically trigger mPTP opening, leading to increased inner membrane permeability, decreased ΔΨm, and ultimately ultrastructural damage (e.g., cristae disruption, membrane fragmentation, and swelling) [93], [94], although JC-1 and TEM do not directly assess mPTP opening. In JC-1 staining, red aggregated fluorescence indicates high ΔΨm and intact function, while enhanced green monomeric fluorescence reflects progressive depolarization and injury [95]. As shown in Fig. 8A, the HIRI group exhibited significantly enhanced green signals and markedly reduced red signals, presenting an overall fluorescence pattern dominated by green, indicating a significant decrease in ΔΨm and pronounced depolarization after IR (quantitative analysis in Fig. 8B). These results are consistent with the elevated Ca^2+^ load and mtROS burst described above, demonstrating that ion homeostasis disruption and amplified oxidative stress during reperfusion have propagated to the level of mitochondrial membrane function, leading to injury progression from "elevated stress" to "functional imbalance". Notably, CPs@HA intervention enhanced red signals and further decreased green signals, yielding a fluorescence distribution closer to that of the Normal group, suggesting its efficacy in restoring ΔΨm stability. We next examined whether this functional recovery was accompanied by restoration of mitochondrial ultrastructure.

**Fig. 8:**
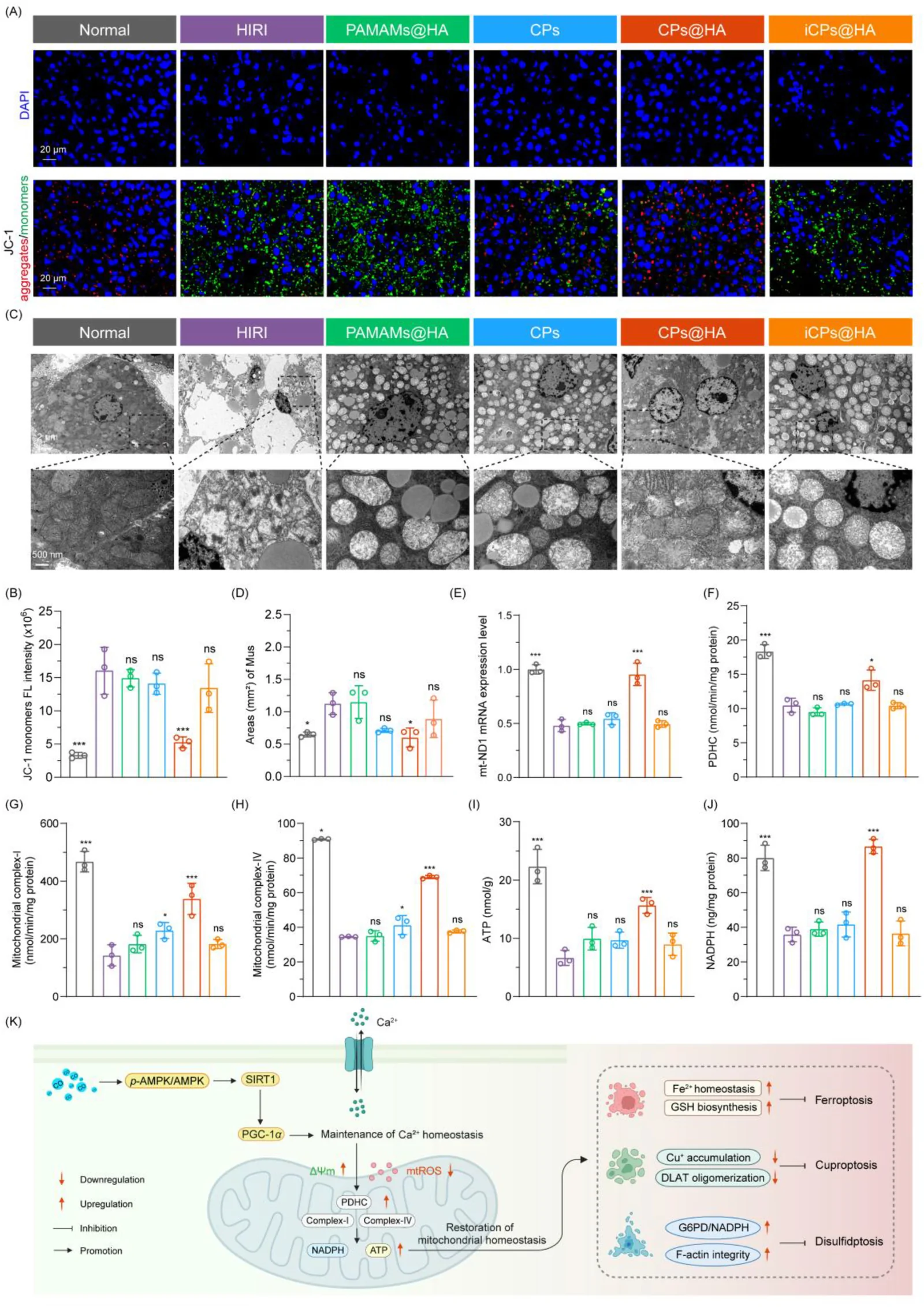
*In vivo* CPs@HA restores mitochondrial membrane potential, ultrastructure, and respiratory chain function, as well as ATP and NADPH levels. (A) Fluorescence imaging of ΔΨm using JC-1 staining in liver tissues (scale bar = 100 μm). (B) Quantitative analysis of JC-1 monomer fluorescence intensity. (C) TEM images of mitochondrial ultrastructure in liver tissues (scale bar = 2 μm and 500 nm). Dashed boxes indicate the magnified regions. (D) Quantitative analysis of the damaged area of mitochondrial ultrastructure. (E) mRNA expression level of mt-ND1 in liver tissues. (F) Activity of PDHC in liver tissues. (G, H) Activities of mitochondrial respiratory chain complex I and complex IV in liver tissues. (I, J) ATP and NADPH levels in liver tissues. (K) Schematic illustration of the mechanism by which CPs@HA reconstructs mitochondrial homeostasis (created with BioRender.com). Statistical analyses were performed by comparing the HIRI group with each of the other groups (*n* = 3). *P < 0.05, **P < 0.01, ***P < 0.001 vs. indicated. ns, not significant.

TEM observation further validated the restorative effect of CPs@HA on mitochondrial damage at the morphological level. As shown in Fig. 8C, hepatocytes in the Normal group exhibited intact mitochondrial morphology with clear structure, continuous membranes, and densely packed, recognizable cristae. In contrast, the HIRI group displayed pronounced ultrastructural damage: mitochondria were generally swollen and rounded with blurred contours, cristae were sparse, fractured, or even absent, and surrounding inflammatory cell infiltration was present. These morphological features indicate that IR has led to significant organic mitochondrial damage, consistent with the observed Ca^2+^ overload, elevated mtROS, and decreased ΔΨm, demonstrating that ion homeostasis disruption and amplified oxidative stress have progressed from functional impairment to structural destruction. Notably, CPs@HA intervention improved mitochondrial ultrastructure: mitochondrial morphology resembled that of the Normal group, with reduced swelling, good membrane continuity, and restored, relatively well-organized cristae. Quantitative analysis of mitochondrial cross-sectional area further confirmed that CPs@HA effectively ameliorated HIRI-induced mitochondrial swelling, reducing it to a level comparable to that of the Normal group (Fig. 8D). Together, these results demonstrate that CPs@HA not only alleviates mitochondrial stress at the molecular and functional levels but also maintains mitochondrial integrity at the structural level, achieving substantial intervention in mitochondrial damage.

To further evaluate the molecular basis of mitochondrial functional recovery, we examined mt-ND1, a mitochondrial DNA-encoded subunit of respiratory chain complex I. mt-ND1 expression reflects the maintenance of mitochondrial gene transcription and is an important indicator of respiratory chain function [96]. As shown in Fig. 8E, mt-ND1 expression was significantly decreased in the HIRI group compared with the Normal group (reduced by 52%), suggesting impaired mitochondrial gene transcription after reperfusion. In contrast, CPs@HA intervention restored mt-ND1 expression to 95.33% of Normal levels, indicating that CPs@HA helps maintain the transcriptional status of respiratory chain-encoding genes while alleviating acute mitochondrial stress, thereby providing a molecular basis for respiratory chain functional reconstruction.

After confirming the restoration of respiratory chain subunit expression (mt-ND1), we next assessed PDHC activity, the upstream substrate input gateway, to evaluate whether improved respiratory chain function was supported by adequate substrate supply. Notably, mt-ND1 and PDHC are located at the respiratory chain end and the substrate supply end of the mitochondrial oxidative metabolic chain, respectively; they are not in a direct upstream-downstream regulatory relationship but are functionally interdependent. Restoration of PDHC activity promotes pyruvate conversion to acetyl-CoA, enhancing TCA cycle substrate supply and providing reducing equivalents (e.g., NADH) for the respiratory chain. Conversely, improved respiratory chain function, particularly complex I, helps maintain PDHC and TCA cycle operation [96], [97], [98]. As shown in Fig. 8F, PDHC activity was significantly decreased in the HIRI group compared with the Normal group (from 18.32 to 10.46 nmol/min/mg protein), indicating impaired pyruvate entry into mitochondrial oxidative metabolism and insufficient TCA cycle substrate supply. PDHC activity was significantly restored after CPs@HA intervention (to 14.14 nmol/min/mg protein), demonstrating improved substrate flux and the stabilization of reducing energy sources.

Consistent with these changes, mitochondrial respiratory chain function was also significantly impaired in the HIRI group, with markedly decreased activities of complex I and complex IV compared with the Normal group (reduced by 69.48% and 61.95%, respectively) (Fig. 8G and H). CPs@HA intervention significantly restored both complex I and IV activities to 72.40% and 75.96% of Normal levels, respectively. These results suggest that CPs@HA ameliorates electron transport chain dysfunction and promotes restoration of oxidative phosphorylation efficiency. Notably, the recovery of complex I activity was highly consistent with the restoration of mt-ND1 expression: the former reflects enzymatic improvement at a key functional node, while the latter indicates maintenance of mtDNA-encoded gene transcription. These mutually corroborating findings support the promoting effect of CPs@HA on respiratory chain functional reconstruction. As the terminal key enzyme of the respiratory chain, restoration of complex IV activity indicates comprehensive recovery rather than improvement at a single node, ultimately manifested as ATP production recovery [99]. This systematic restoration of mitochondria, from molecular and structural to metabolic function, culminates in improved energy status. IR injury significantly impaired mitochondrial energy production, whereas CPs@HA intervention led to a marked recovery in ATP levels (increased by 2.36-fold compared with the HIRI group, reaching 70.12% of Normal levels, Fig. 8I), indicating effectiveness in alleviating the energy crisis. Concurrently, NADPH levels were also significantly restored after CPs@HA intervention, approaching Normal levels (Fig. 8J). The simultaneous improvement of ATP and NADPH is significant: ATP reflects oxidative phosphorylation and energy supply, while NADPH represents a key reducing equivalent for antioxidant capacity and disulfide bond reduction.

Taken together, CPs@HA, through upstream activation of the AMPK–SIRT1–PGC-1*α* energy-sensing axis, subsequently inhibits the vicious cycle of Ca^2+^ overload and mtROS elevation during early reperfusion, maintains ΔΨm and mitochondrial structural integrity, and restores PDHC-TCA substrate input and respiratory chain complex activity, ultimately achieving simultaneous recovery of ATP energy and NADPH reducing power. Thus, CPs@HA restores mitochondrial homeostasis and exhibits consistent protective advantage across multiple mitochondrial parameters (Fig. 8K). In contrast, the PAMAMs@HA, CPs, and iCPs@HA groups showed only limited overall improvement, with effects consistently weaker than those of CPs@HA. This stable intergroup difference suggests that the mitochondrial protective effect of CPs@HA primarily relies on HA-mediated active targeting and on-demand CO release from CORM-401 within the HIRI lesion microenvironment, rather than the PAMAMs carrier alone, non-targeted delivery (CPs), or inactivated CO (iCPs@HA). The restoration of mitochondrial homeostasis serves as the common mechanistic basis for the synergistic inhibition of the three types of programmed cell death. First, NADPH recovery helps maintain GPX4-mediated lipid peroxide scavenging, thereby reducing ferroptosis susceptibility [61]. Second, improved PDHC complex and respiratory chain function alleviates mitochondrial metabolic stress and redox collapse, and together with copper homeostasis restoration, reduces key cuproptosis events such as abnormal DLAT oligomerization [100]. Third, as the core reducing equivalent for maintaining disulfide bond reduction and sulfhydryl homeostasis, NADPH recovery provides necessary conditions for inhibiting glucose starvation-related disulfide stress and F-actin collapse, thereby weakening the triggering basis for disulfidptosis [59]. Therefore, when mitochondrial energy and reducing power are restored and oxidative stress is buffered, cellular susceptibility to ferroptosis, cuproptosis, and disulfidptosis collectively decreases.

With the reconstruction of mitochondrial homeostasis, excessive ROS and the lipid peroxidation it triggers are inhibited, and the cellular redox environment tends toward balance, thereby attenuating inflammatory responses and improving liver tissue damage outcome. Thus, alongside mitochondrial recovery, CPs@HA exhibits protective effects associated with attenuation of the three-cell death-related processes, mutually corroborating the previously described improvements in liver function, alleviation of oxidative stress and inflammation, and the overall trend of transcriptome normalization *in vivo*.

## 3 Conclusions

In summary, we have developed a CO-releasing nanoformulation (CPs@HA) that attenuates three oxidative stress-linked programmed cell death pathways, namely ferroptosis, cuproptosis, and disulfidptosis, in HIRI. In a mouse model, CPs@HA alleviates hepatocellular damage and restores liver function. Mechanistically, our findings link mitochondrial dysfunction to changes associated with these cell death pathways. CPs@HA restores mitochondrial homeostasis by activating the AMPK–SIRT1–PGC-1α axis, restoring Ca^2+^ homeostasis, preserving mitochondrial integrity, and recovering respiratory chain activity, ATP synthesis and NADPH reducing capacity. This is accompanied by interruption of the vicious cycle of oxidative stress, metabolic collapse, and cell death, suggesting a potentially integrated therapeutic mechanism. Taken together, this study provides an integrated framework connecting pathological-microenvironment-responsive CO delivery with hepatoprotection, mitochondrial recovery, and the modulation of multiple cell-death processes.

Several limitations of this study should be acknowledged. In the present study, CPs@HA was administered before ischemia to evaluate its prophylactic efficacy. This regimen is clinically relevant to settings in which hepatic ischemia can be anticipated, such as liver transplantation and hepatic resection. Because hepatic IRI involves local ischemic injury followed by inflammation-mediated reperfusion injury, pretreatment ensured that CPs@HA was present throughout both phases and enabled early intervention before the injury cascade was fully established [82]. Nevertheless, the absence of direct comparisons among pre-ischemic, intraoperative, and post-ischemic administration represents a limitation of the present study. Future studies should compare CPs@HA administration before ischemia, at reperfusion, and at defined intervals thereafter to determine its optimal timing and post-injury therapeutic potential. Although CPs@HA treatment was accompanied by coordinated changes in the AMPK–SIRT1–PGC-1α axis, Ca^2+^ homeostasis, mitochondrial redox status, bioenergetics, and multiple cell-death-associated markers, the present study was primarily designed to characterize these molecular and functional associations rather than to establish a complete causal hierarchy. Pharmacological inhibition or genetic loss-of-function approaches targeting AMPK, SIRT1, PGC-1α, or other key mitochondrial regulators will therefore be required to determine whether these pathways are indispensable for the protective effects of CPs@HA. Additionally, the involvement of ferroptosis, cuproptosis, and disulfidptosis was supported by transcriptomic signatures, biochemical indicators, molecular markers, and complementary pathway-oriented cellular stress models. However, these findings do not establish that each form of regulated cell death independently or obligatorily contributes to HIRI. Future studies incorporating pathway-specific pharmacological rescue and genetic interventions directly in HIRI models will be necessary to clarify their individual contributions. Although the coordinated recovery of mitochondrial function and attenuation of ferroptosis-, cuproptosis-, and disulfidptosis-associated features suggest that mitochondrial homeostasis may represent a convergent regulatory node, the present data do not formally establish mitochondria as a common upstream causal hub. Targeted mitochondrial perturbation studies will be needed to define the temporal and causal relationship between mitochondrial dysfunction and these cell-death-associated processes. Finally, RNA-seq and GSEA analyses in this study should be interpreted as identifying treatment-associated transcriptional signatures and candidate pathways rather than as independent evidence of pathway activation or causality. Future studies integrating pathway-specific functional interventions with transcriptomic and molecular analyses will help further delineate the mechanistic basis of CPs@HA-mediated protection.

## 4 Experimental section

### 4.1 Materials

Amino-terminated generation 3 PAMAM dendrimers were purchased from Weihai CY Dendrimer Tech. Co., Ltd. (China). DSP and LPS were obtained from Sigma-Aldrich (USA). HA (Mw 500,000 Da) was purchased from Bloomage Freda (Shandong, China). CORM-401 and FL-CO-1 were synthesized in our laboratory. ABTS, DPPH, and H_2_O_2_ scavenging assay kits were purchased from Beijing Bioss Biotech. Co., Ltd. (China). FBS was from Gibco (USA). Cell culture dishes and flasks were obtained from Wuxi NEST Biotech. Co., Ltd. (China). Calcein-AM/PI double staining kit, Hoechst 33342, hematoxylin and eosin (H&E) staining reagent, and Cell Counting Kit-8 (CCK-8) were purchased from Nanjing KeyGen Biotech. Co., Ltd. (China). Cysteine, ATP, NADPH, mitochondrial complex I and IV activity assay kits, PDH activity assay kit, phalloidin dye, glass-bottom culture dishes, and BCA protein quantification kit were from Beyotime Biotech. (China). JC-1 fluorescent probe, ROS detection probe (DCFH-DA), MitoTracker Red CMXRos, and Cy5.5-NHS were purchased from Solarbio (USA). ROS Green H_2_O_2_ probe was provided by Shanghai Maokang Biotech. Co., Ltd. (China). MitoSOX dye and Rhod-2 calcium dye were obtained from Thermo Fisher (USA). Elesclomol was purchased from TargetMol Chemicals Inc (USA). RSL3 and CuCl_2_·2H_2_O were provided by Beijing Push Biotech. Co., Ltd. (China). Copper ion assay kit, and detection kits for SOD, CAT, GSH, GSH-Px, GSSG, MDA, and ferrous ions were all purchased from Suzhou Grace Biotech. Co., Ltd. (China). GPX4 ELISA kit was obtained from Shanghai ZCIBIO Tech. Co., Ltd. (China). Mouse COHb ELISA kit was purchased from Beijing Meimian Biotech. Co., Ltd. (China). Mouse macrophage cell line RAW264.7 and human hepatocellular carcinoma cell line HepG2 were provided by Wuhan Procell Life Tech. Co., Ltd. (China). Antibodies against DLAT, G6PD, FLNA, *p*-AMPK*α*, AMPK*α*, SIRT1, PGC-1*α*, CD86, HO-1, CD163, 8-OHdG, iNOS, and GPX4, as well as mouse IL-4, IL-10, and IL-6 ELISA kits, were purchased from Proteintech (USA). Antibodies against SLC7A11, GCLM, GCLC, FTH1, FTL, PCBP1, NCOA4, and LC3 were purchased from Shanghai Yeasen Biotech. Co., Ltd. (China).

### 4.2 Preparation and characterization of CPs

PAMAM dendrimers (20 mg) were dissolved in 4 mL of DMF. Then, 2 mL of DMF containing 10 mg of DSP was added dropwise. The mixture was stirred at room temperature for 24 h, followed by thorough dialysis against DMF and subsequently against deionized water (Spectrum, MWCO 1000 Da). The dialyzed product was lyophilized to obtain crosslinked PAMAMs as a flocculent powder.

CORM-401 dissolved in methanol was added dropwise to a methanol solution of the PAMAMs at a mass ratio of 1/1. The mixture was magnetically stirred in the dark at room temperature for 24 h. The resulting solution was dialyzed against deionized water for 24 h (Spectrum, MWCO 1000 Da) and then lyophilized to yield a pale-yellow powder, designated as CORM-401@PAMAMs (CPs).

The particle size, size distribution, zeta potential, and morphology of CPs were determined using a Zetasizer Nano ZS90 instrument (Malvern Panalytical Ltd. Malvern, Worcestershire, UK) and transmission electron microscopy (JEM-2100 Plus, JEOL Ltd. Tokyo, Japan). For storage stability evaluation, CPs (1 mg/mL in PBS, pH 7.4) were incubated at 4 °C, and changes in solution color were observed over 7 days. To assess serum stability, CPs (1 mg/mL) were incubated in 20% FBS at 37 °C, and their particle size and zeta potential were continuously measured over 48 h.

### 4.3 Preparation and characterization of CPs@HA and iCPs@HA

CPs (1 mg/mL) and HA (1 mg/mL) were separately dispersed in deionized water. Equal volumes of the two solutions were then mixed at a mass ratio of 1/1 and vortexed at maximum speed for 5 min using a vortex mixer (Vortex-Genie 2, Scientific Industries, Bohemia, New York, USA). The reaction mixture was stirred magnetically in the dark at room temperature for 24 h, dialyzed against deionized water for 24 h (Spectrum, MWCO 1000 Da), and lyophilized to obtain a pale-yellow powder, designated as CPs@HA.

The particle size, zeta potential, and morphology of CPs@HA were determined using DLS and TEM. Elemental mapping was performed to analyze the distribution of CORM-401 within CPs@HA. For storage stability evaluation, CPs@HA (1 mg/mL) in PBS (pH 7.4) were incubated at 4 °C for 7 days, and changes in solution color, particle size, zeta potential, and PDI were monitored. To assess stability under blood circulation-mimicking conditions, CPs@HA (1 mg/mL) were incubated in PBS containing 15% FBS at 37 °C for 48 h, and the same parameters were recorded.

The Mn content in CPs@HA was measured by ICP-OES (Optima 7300DV, PerkinElmer, Waltham, Massachusetts, USA) to determine drug loading. Based on the Mn content, the loading content and encapsulation efficiency of CORM-401 in CPs@HA were calculated. UV-vis absorption spectroscopy (UH5700, Hitachi High-Tech Corporation, Tokyo, Japan) was used to confirm the characteristic absorption peak of encapsulated CORM-401.

iCPs@HA was prepared by incubating CPs@HA with an equimolar amount of H_2_O_2_ relative to the loaded CORM-401 at 37 °C for 24 h to exhaust CO release. The product was then dialyzed against ddH_2_O for 24 h and lyophilized. The absence of further CO release was confirmed using a CO detector.

### 4.4 ROS-responsive CO release and ROS scavenging performance of CPs@HA

The ROS-responsive CO release behavior of CPs@HA was evaluated using a custom-designed CO detection system. Briefly, 2 mL of CPs@HA solution (2 mg/mL) and a CO detector (BH-90, Baoshian Electronic Tech. Co., Zhengzhou, Henan, China) were placed in a cylindrical glass container, and the system was sealed. Then, 2 mL of H_2_O_2_ solution at different concentrations (0, 0.2, 2, and 20 mmol/L) was injected into the CPs@HA solution using a syringe to trigger CO release. The CO gas concentration (ppm) was recorded at each time point, and the total amount of CO released was calculated according to a previously reported formula [22].

The scavenging capacity of CPs@HA against H_2_O_2_, ABTS·^+^, and DPPH· was assessed following the instructions of commercial kits. Specifically, 10 μL of CPs@HA at different concentrations (75 and 150 μg/mL) was separately reacted with a solution containing approximately 100 μmol/L H_2_O_2_, a pre-prepared ABTS·^+^ working solution, or a DPPH· working solution. For H_2_O_2_ and ABTS·^+^, the reaction was allowed to proceed for 5–15 min; for DPPH·, the reaction time was 0.5–1.5 h. The absorbance was then measured at the wavelength specified in the kit instructions, and the radical scavenging activity was calculated according to the provided formula.

### 4.5 Cell culture and cytotoxicity evaluation

RAW264.7 and HepG2 cells were cultured in their respective complete media (Wuhan Pricella Biotech Co., Ltd. CM-0190, Wuhan, China). “Normal macrophages” refer to RAW264.7 cells cultured without LPS stimulation, whereas “activated macrophages” were obtained by treatment with 3 μg/mL LPS for 24 h.

The cytotoxicity of CPs@HA against RAW264.7 and HepG2 cells was evaluated using the CCK-8 assay and live/dead staining. Briefly, cells were seeded into 96-well plates at 1 × 10^4^ cells/well and cultured overnight. The medium was then replaced with fresh medium containing CPs@HA (50–250 μg/mL), and the cells were incubated for another 24 h. After washing with PBS, the cells were stained with Calcein-AM (5 μmol/L) and propidium iodide (PI, 5 μmol/L) for visualization under an inverted fluorescence microscope (AxioObserver3, Carl Zeiss, Jena, Thuringia, Germany). For the CCK--8 assay, cells were washed with PBS, incubated with 10 μL of CCK-8 solution for 1 h, and the absorbance at 450 nm was measured using a microplate reader. Cells treated with PBS or CPs (120 μg/mL) served as controls in both assays. Based on cytocompatibility and functional screening, CPs@HA at 75 and 150 μg/mL was selected for subsequent assays, with 150 μg/mL used for mechanistic studies. Based on the CORM-401 loading contents (Tables S1–S2), CPs at 120 μg/mL was used to match the CORM-401 concentration of CPs@HA at 150 μg/mL.

### 4.6 Hemocompatibility evaluation of CPs@HA

The hemocompatibility of CPs@HA was assessed using a standard hemolysis test. Whole blood was collected from healthy Sprague–Dawley mice *via* retro-orbital bleeding and centrifuged at 3000 rpm for 15 min to isolate red blood cells (RBCs). The RBC pellet was resuspended in PBS to obtain an RBC suspension. Aliquots (500 μL) of the RBC suspension were incubated with PBS (negative control), PAMAMs (150 μg/mL), CPs (120 μg/mL), CPs@HA (75 or 150 μg/mL), or Triton X-100 (positive control). All treatments were performed in triplicate. After incubation at 37 °C for 1 h, the samples were centrifuged at 2000 ×*g* for 10 min at 4 °C, and the supernatants were collected. The absorbance (OD) at 540 nm was measured using a microplate reader. The hemolysis rate was calculated as follows: Hemolysis (%) = (OD_sample_ – OD_PBS_)/(OD_Triton_ – OD_PBS_) × 100%.

### 4.7 Cellular uptake

To prepare Cy5.5-labeled nanogels, CPs@HA (1 mg/mL) was mixed with Cy5.5 NHS ester (25 μg in 25 μL DMSO) and stirred at room temperature for 4 h. The product was then dialyzed (Spectrum, MWCO 1000 Da) and lyophilized. For uptake experiments, activated macrophages and HepG2 cells were seeded in culture dishes at 4 × 10^5^ cells/dish and cultured for 24 h. The cells were then incubated with DMEM containing 150 μg/mL Cy5.5-CPs@HA for 2 h. After washing with PBS, the cells were visualized by confocal laser scanning microscopy (CLSM, LSM 700, Carl Zeiss AG, Jena, Germany). Controls included normal macrophages treated with 150 μg/mL CPs@HA, activated macrophages treated with 120 μg/mL Cy5.5-CPs, and activated macrophages pre-incubated with HA (5 mg/mL) before exposure to Cy5.5-CPs@HA.

### 4.8 Intracellular CO imaging

Intracellular CO generation was monitored using a CO probe system (PdCl_2_ + FL-CO-1). Activated macrophages were seeded in culture dishes at 4 × 10^5^ cells/dish and cultured for 24 h. The cells were then incubated with DMEM containing 75 or 150 μg/mL CPs@HA for 4 h. After removing the medium, the cells were treated with 0.3 mL of serum-free DMEM containing 5 μmol/L Hoechst 33342, 5 μmol/L FL-CO-1, and 5 μmol/L PdCl_2_ for 0.5 h. Following two washes with PBS, intracellular CO generation was visualized by CLSM. Controls included normal macrophages, untreated activated macrophages, and activated macrophages treated with CPs (120 μg/mL). Fluorescence intensities were quantified using ImageJ software.

### 4.9 In vitro antioxidant and anti-inflammatory properties of CPs@HA

The scavenging effects of CPs@HA on intracellular ROS and H_2_O_2_ in activated macrophages were detected using DCFH-DA and ROS Green H_2_O_2_ probes, respectively. Cells were seeded in culture dishes at 2 × 10^5^ cells/well and cultured for 24 h, then exposed to DMEM containing CPs@HA (150 μg/mL) for another 24 h. After removing the medium, the cells were incubated for 0.5 h with 0.3 mL of solution containing 5 μmol/L Hoechst 33342 and either 5 μmol/L DCFH-DA or 5 μmol/L ROS Green H_2_O_2_. Following washing with PBS, intracellular fluorescence was visualized by inverted fluorescence microscopy. Excitation/emission wavelengths were 644/665 nm for DCFH-DA and 490/514 nm for ROS Green H_2_O_2_. Controls included PBS-treated normal macrophages, untreated activated macrophages, and activated macrophages treated with CPs (120 μg/mL) or CPs@HA (75 μg/mL).

The antioxidant capacity of CPs@HA was assessed by measuring CAT, SOD, and GSH-Px activities using commercial kits. Activated macrophages (1 × 10^7^ cells/dish) were cultured for 24 h and then incubated with DMEM containing 150 μg/mL CPs@HA for 24 h. Cells were collected, lysed by ultrasonication on ice (300 W, 5 s on/25 s off, 30 min), and centrifuged at 12,000 ×*g* for 15 min at 4 °C. The supernatants were collected for protein quantification using the BCA assay. Aliquots were mixed with reagents according to the kit instructions, and absorbance was measured: CAT at 510 nm (H_2_O_2_ decomposition), SOD at 450 nm (pyrogallol autoxidation inhibition), and GSH-Px at 412 nm (NADPH oxidation). Activities were calculated from standard curves and normalized to total protein.

To assess HO-1 expression, activated macrophages (2 × 10^5^ cells/well in 6-well plates) were cultured for 24 h and then treated with DMEM containing 150 μg/mL CPs@HA for 24 h. Cells were collected for IF staining using an HO-1 antibody and observed by an inverted fluorescence microscope. HO-1 mRNA levels were quantified by RT-qPCR. mRNA was extracted, reverse transcribed, amplified, and analyzed using a real-time PCR system. Primer sequences are listed in Supporting Information Table S5.

To evaluate phenotypic changes, activated macrophages seeded in confocal dishes at 2 × 10^5^ cells/well were cultured for 24 h and then treated with DMEM containing 150 μg/mL CPs@HA for 24 h. Cell morphology was observed by inverted fluorescence microscopy, and cell elongation and the number of protrusions were quantified. Cells were immunostained with iNOS and CD206 antibodies and visualized by CLSM. mRNA levels of iNOS, TNF-*α*, IL-1*β*, CD206, Arg-1, and IL-4 were quantified by RT-qPCR. Controls included normal macrophages, untreated activated macrophages, and activated macrophages treated with CPs (120 μg/mL) or CPs@HA (75 μg/mL). Primers are listed in Table S5.

### 4.10 Evaluation of therapeutic effects of CPs@HA in a mouse model of HIRI

All animal experiments were approved by the Animal Welfare and Ethics Committee of Wenzhou Medical University (approval No. wydw2026-0169) and conducted in accordance with laboratory animal care guidelines. Male ICR mice (6–8 weeks old) were purchased from Beijing SPF Biotech. Co., Ltd. (China) and housed under standard conditions (22 °C, 30–70% humidity, 12 h light/dark cycle) with free access to food and water.

Prior to modeling, mice were fasted for 12 h, anesthetized with isoflurane, and placed on a heated surgical pad. After abdominal disinfection with povidone-iodine, a midline incision was made to expose the hepatic portal area. The hepatic artery, portal vein, and bile duct were clamped with a vascular clip to induce approximately 70% hepatic ischemia, confirmed by liver pallor within 30 s. After 60 min of ischemia, the clip was removed to restore blood flow, and the abdomen was sutured. Sham group mice underwent the same surgical procedures as the HIRI group, except that the hepatic portal triad was exposed but not clamped. To determine the effective dose range, CPs@HA (0.25, 0.5, 1.0, 1.5, and 2.0 mg/mL) was administered *via* the tail vein 2 h before ischemia. Mice were euthanized 24 h after reperfusion, and the optimal dose was assessed based on liver function (AST and ALT) and COHb levels. Serum ALT and AST were selected as established biochemical indicators of hepatocellular damage and acute liver injury severity. Based on the maximal reductions in these markers without an increase in COHb, 1.5 mg/mL (0.30 mg per mouse) was selected for subsequent experiments.

For liver-targeting assessment, HIRI mice received Cy5.5-labeled CPs@HA (1.5 mg/mL, 200 μL) *via* the tail vein. Hepatic distribution was monitored using an *in vivo* imaging system (IVIS Lumina XRMS Series III, PerkinElmer, Waltham, Massachusetts, USA) at 0, 2, 4, 8, 12, and 24 h post-injection.

To evaluate therapeutic potential, mice were randomly divided into 7 groups (n = 5): (1) Normal (healthy), (2) Sham, (3) HIRI, (4) PAMAMs@HA + HIRI, (5) CPs + HIRI, (6) CPs@HA + HIRI, and (7) iCPs@HA + HIRI. The Normal, Sham, and HIRI groups received 200 μL of saline *via* the tail vein 2 h before surgery; the other groups received 200 μL of PAMAMs@HA (1.5 mg/mL), CPs (1.2 mg/mL), CPs@HA (1.5 mg/mL), or iCPs@HA (1.5 mg/mL). At 24 h after HIRI induction, retro-orbital blood was collected for serum ALT, AST, and BUN assays. Mice were then euthanized, and the left liver lobes were collected for histopathology. Liver tissues were washed with PBS, fixed in formalin, embedded in paraffin, sectioned, and stained with TUNEL and H&E.

Hepatic CO generation was monitored using a CO probe system (PdCl_2_ + FL-CO-1). Fresh liver tissues were embedded in OCT, frozen in liquid nitrogen, and cryosectioned. Sections were rehydrated in PBS, washed, and stained with FL-CO-1 (5 μmol/L) and PdCl_2_ (5 μmol/L) for 30 min in the dark. After washing with PBS, sections were lightly fixed with 4% paraformaldehyde, mounted with an antifade medium, and observed by CLSM.

To assess antioxidant and anti-inflammatory effects, liver sections were incubated with anti-8-OHdG, anti-CD86, and anti-CD206 antibodies, followed by secondary antibody incubation, and visualized by CLSM. For antioxidant enzyme activity assays, liver tissue was homogenized in 2 mL of cold PBS, centrifuged (10,000 rpm, 4 °C, 10 min), and CAT, SOD, and GSH-Px activities were measured using commercial kits. Cytokine levels (IL-1*β*, IL-4, IL-6, and IL-10) in the homogenates were determined by ELISA.

RNA-seq was commissioned to Shanghai Personal Biotech. Co., Ltd., including mRNA enrichment, library preparation, and sequencing. High-quality RNA-seq data were obtained following established protocols [22], [101]. To systematically evaluate transcriptional changes in regulated cell death pathways, five major modalities, ferroptosis, cuproptosis, disulfidptosis, pyroptosis, and senescence, were quantified using a gene set-based scoring strategy. For each modality, a curated set of gene features was constructed based on previous reports and categorized into positive (pro-death) and negative (anti-death) regulators [102], [103], [104], [105], [106]. Single-sample Gene Set Enrichment Analysis (ssGSEA) was performed using the GSVA package to calculate enrichment scores for each gene set per sample. For each death modality, two scores were obtained per sample: ssGSEA scores for the positive and negative regulator sets. To quantify pathway activity, DPI was calculated as: DPI = Enrichment_positive_ − Enrichment_negative_. This bidirectional scoring system reflects the balance between pro-death and anti-death transcriptional programs, with a higher DPI indicating enhanced activation of the corresponding pathway. All analyses were performed in R using the GSVA, ggplot2, and ggpubr packages. Data and code are available at GitHub (https://github.com/814QiangZhou227/Xiaojun-Cai).

To evaluate the intervention effects of CPs@HA on multiple cell death pathways, levels of MDA, GSSG, and Fe^2+^ were first measured in liver homogenates. Liver sections were then immunostained with anti-GPX4 antibody and visualized by CLSM. GPX4 expression was further quantified by ELISA to assess anti-ferroptosis effects. Meanwhile, Cu^+^ content in liver homogenates was measured using a kit, and the cuproptosis key protein DLAT was analyzed by immunofluorescence. Additionally, cysteine levels in the homogenates were determined, and F-actin cytoskeletal changes were observed by CLSM after phalloidin staining (30 min in the dark).

To assess biosafety, retro-orbital blood was collected at 2, 4, 8, 12, and 24 h post-injection. Plasma was separated by centrifugation for COHb ELISA. Whole blood was used for hematological analysis (white blood cells, WBC; platelets, PLT; hemoglobin, Hb; red blood cells, RBC). Major organs (heart, liver, spleen, lungs, and kidneys) were collected for H&E staining.

### 4.11 Molecular mechanism of ferroptosis inhibition by CPs@HA

To evaluate the protective effect of CPs@HA against RSL3-induced ferroptosis in HepG2 cells, CCK-8 assay and live/dead staining were used. For RSL3 concentration optimization, HepG2 cells were seeded in 96-well plates at 1 × 10^4^ cells/well, cultured overnight, and then treated with RSL3 (0–64 μg/mL) for 24 h, followed by cell viability assessment.

For protection assessment, HepG2 cells seeded in 96-well plates at 1 × 10^4^ cells/well overnight were treated with RSL3 (8 μg/mL) supplemented with CPs@HA (150 μg/mL) for 24 h. Cell viability was measured by CCK-8 assay at 450 nm after washing with PBS. Live/dead staining (Calcein-AM/PI) was observed by inverted fluorescence microscopy.

RNA sequencing was performed on cells subjected to different treatments to investigate the effects of CPs@HA on the ferroptosis-related transcriptome in RSL3-induced HepG2 cells. For specific procedures, refer to the preceding section “Evaluation of Therapeutic Effects of CPs@HA in a Mouse Model of HIRI”. Data and code are available at GitHub (https://github.com/814QiangZhou227/Xiaojun-Cai).

For WB, cells were lysed with RIPA buffer containing protease inhibitors. Equal amounts of protein were separated by SDS-PAGE, transferred to membranes, and probed with antibodies against FTH1, FTL, PCBP1, SLC7A11, GCLM, GCLC, NCOA4, and LC3. Signals were visualized by enhanced chemiluminescence (ECL) and quantified using ImageJ software, with *β*-actin as the loading control.

Levels of MDA, GSH, GSSG, and Fe^2+^ were measured using commercial kits. HepG2 cells (3 × 10^6^ cells/flask) treated with RSL3 + CPs@HA (150 μg/mL) for 24 h were lysed by ultrasonication on ice (300 W, 5 s on/25 s off, 30 min). The supernatants were collected for protein quantification using the BCA assay and then mixed with detection reagents. Absorbance was measured at 532 nm (MDA), 600 nm and 412 nm (GSSG/GSH), and 562 nm (Fe^2+^). Concentrations were calculated from standard curves and expressed as MDA (pmol/10^4^ cells), GSH and GSSG (μmol/10^4^ cells), and Fe^2+^ (μg/10^4^ cells). GPX4 levels (pg/mL) were quantified by ELISA.

Intracellular ROS was detected by flow cytometry using the DCFH-DA probe. Treated cells were incubated with 10 μmol/L DCFH-DA for 30 min at 37 °C in the dark, then washed, resuspended, and analyzed. Data are expressed as the percentage of ROS-positive cells normalized to controls. Controls included normal cells and RSL3-treated cells.

### 4.12 Molecular mechanism of cuproptosis inhibition by CPs@HA

To determine the optimal working concentrations of CuCl_2_ and ES, HepG2 cells were seeded in 96-well plates at 1 × 10^4^ cells/well, cultured overnight, and then treated with various concentrations of ES (0.1–100 μmol/L) or CuCl_2_ (0–160 nmol/L) for 24 h. After washing with PBS, absorbance was measured at 450 nm to assess cell viability. Following the determination of the optimal concentration for each drug, cells were treated with different ratios of ES to CuCl_2_ (ranging from 1/1 to 1/1000) for 24 h, and cell viability was assessed by CCK-8 assay.

To evaluate the protective effect of CPs@HA against ES/Cucl_2_-induced cuproptosis, HepG2 cells (1 × 10^4^ cells/well) seeded overnight were treated with 100 nmol/L ES supplemented with 1 μmol/L CuCl_2_ and 150 μg/mL CPs@HA for 24 h. Cell viability was then assessed by CCK-8 assay. Live/dead staining was performed using Calcein-AM/PI. DLAT expression was analyzed by immunofluorescence staining and observed by CLSM.

RNA-seq was performed on both ES/CuCl_2_-treated and ES/CuCl_2_ + CPs@HA-treated cells to assess transcriptomic changes. Differential expression analysis was conducted to identify significantly altered genes between the two groups, using the criteria of |log_2_FC| > 0.5 and FDR < 0.05. The identified DEGs were subsequently subjected to GO and KEGG enrichment analyses to explore their associated biological functions and pathways. In addition, GSEA was performed to evaluate transcriptome-wide pathway alterations. Furthermore, a gene set-based scoring strategy was applied to quantitatively assess cuproptosis at the transcriptomic level. Data and code are available at GitHub (https://github.com/814QiangZhou227/Xiaojun-Cai).

Intracellular levels of Cu^+^, GSSG, GSH, ATP, PDH, and NADPH were measured using commercial kits. ES/CuCl_2_-induced HepG2 cells (3 × 10^6^ cells/flask) cultured for 24 h were treated with ES/CuCl_2_ medium containing CPs@HA (150 μg/mL) for another 24 h. Cells were then lysed by ultrasonication on ice (300 W, 5 s on/25 s off, 30 min). After centrifugation, the supernatants were collected for protein quantification using the BCA assay and then mixed with the corresponding detection reagents. Absorbance was measured at 600 nm (GSSG), 412 nm (GSH), 483 nm (Cu^+^), 570 nm (ATP), 450 nm (PDH), and 340 nm (NADPH), and target levels were calculated using the kit formulas.

Intracellular ROS was detected by flow cytometry using the DCFH-DA probe as described above. Controls included normal cells and ES/CuCl_2_-treated cells.

### 4.13 Molecular mechanism of disulfidptosis inhibition by CPs@HA

To determine the optimal glucose starvation duration, HepG2 cells were seeded in 96-well plates at 1 × 10^4^ cells/well, cultured overnight, and then incubated in glucose-free medium (DMEM without d-glucose, supplemented with 10% FBS and 1% penicillin–streptomycin) for different periods (0, 12, 24, 36, and 48 h). After washing with PBS, cell viability was assessed by CCK-8 assay at 450 nm.

To evaluate the protective effect of CPs@HA against glucose starvation (Glu(−))-induced disulfidptosis, HepG2 cells (1 × 10^6^ cells/well) were seeded in 6-well plates and cultured for 24 h. Subsequently, the cells were incubated for 24 h in glucose-free medium containing CPs@HA (150 μg/mL). Cell viability was then assessed by CCK-8 assay.

RNA-seq was performed on both Glu(−)-treated and Glu(−) + CPs@HA-treated cells to assess transcriptomic changes. Differential expression analysis was conducted using the criteria |log_2_FC| > 0.5 and FDR < 0.05. GO and KEGG enrichment analyses were performed on the selected DEGs to identify significantly associated biological processes and signaling pathways. GSEA was subsequently performed to capture global pathway alterations. To specifically assess the regulation of disulfidptosis, a gene set-based scoring strategy was employed to quantitatively evaluate disulfidptosis-related activity at the transcriptomic level. Data and code are available on GitHub (https://github.com/814QiangZhou227/Xiaojun-Cai).

To evaluate the protective effect of CPs@HA against glucose starvation-induced disulfidptosis, cells were first treated as described above; the cytoskeleton was then stained with phalloidin and visualized by CLSM. Intracellular levels of Cys, GSSG, GSH, ATP, and NADPH were measured using commercial kits. Absorbance for Cys was recorded at 412 nm, and Cys levels were calculated from an l-cysteine standard curve, expressed as nmol/mg protein. Other indicators were determined according to the methods described above. FLNA and G6PD expression were analyzed by Western blotting following the same procedures as described above. Intracellular ROS was detected by flow cytometry using the DCFH-DA probe as described above. Controls included untreated and Glu(−)-treated cells.

### 4.14 Mitochondrial protective effects of CPs@HA

To systematically evaluate the mitochondrial protective effects of CPs@HA, mice were randomly assigned to six groups (n = 5): (1) Normal (healthy control), (2) HIRI, (3) iCPs@HA + HIRI, (4) PAMAMs@HA (1.5 mg/mL) + HIRI, (5) CPs (1.2 mg/mL) + HIRI, and (6) CPs@HA (1.5 mg/mL) + HIRI. All treatments and procedures were performed as described above.

To further elucidate the CO-mediated mitochondrial protective effects, GSEA-GO enrichment analysis was performed on RNA-seq data from liver tissues of the HIRI and CPs@HA + HIRI groups to identify mitochondrial-related pathways and key regulatory molecules. Subsequent mechanistic studies focused on mitochondrial function and redox homeostasis, and the results were further integrated into regulatory networks associated with ferroptosis, cuproptosis, and disulfidptosis.

To evaluate mitochondrial protection by CPs@HA, mouse liver tissues collected from *in vivo* experiments were first treated as described above and then subjected to Western blotting to determine the protein levels of *p*-AMPK*α*, AMPK*α*, SIRT1, and PGC-1*α*.

To assess mitochondrial Ca^2+^ homeostasis and mitochondrial integrity *in vivo*, liver sections from each group were incubated with 5 μmol/L Rhod-2 and 100 nmol/L MitoTracker for 30 min in the dark, washed, further incubated in dye-free buffer for 30 min in the dark, then fixed with 4% paraformaldehyde, mounted, and imaged by CLSM. To assess mtROS levels, sections were stained with 5 μmol/L MitoSOX for 15 min at 37 °C in the dark, washed, fixed, mounted, and imaged similarly. To assess changes in ΔΨm, fresh liver tissue sections were stained with JC-1 working solution for 30 min at 37 °C in the dark, washed three times with PBS, and immediately imaged by CLSM.

For mitochondrial ultrastructure observation, frozen liver sections were processed for TEM, including chemical fixation, post-fixation, gradient dehydration, resin embedding, ultrathin sectioning, and heavy metal staining. Images were acquired by TEM, and mitochondrial cross-sectional area was quantified using ImageJ software. mt-ND1 expression was analyzed by RT-qPCR. Levels of ATP, NADPH, and mitochondrial respiratory enzymes (complex I, complex IV, and PDHC) were measured using commercial kits.

### 4.15 Statistical analysis

All data are presented as the mean ± standard deviation (SD) and were obtained from at least three independent experiments. Statistical analysis was performed using unpaired Student's t-test or one-way analysis of variance (ANOVA), as appropriate. Data were analyzed using Prism software (version 10; GraphPad, La Jolla, CA, USA). A value of *P* < 0.05 was considered statistically significant. Significance levels are indicated as *P < 0.05, **P < 0.01, and ***P < 0.001; ns: not significant.

## CRediT authorship contribution statement

**Jiayu Li:** Formal analysis, Investigation, Methodology, Validation, Visualization, Writing – original draft, Data curation. **Mengzhu Xu:** Investigation, Methodology, Software, Formal analysis, Data curation. **Qiang Zhou:** Project administration, Visualization, Writing – review & editing. **Hanjing Ni:** Investigation, Methodology. **Chengge Shi:** Visualization, Formal analysis. **Tianci Zuo:** Investigation, Methodology. **Lingjun Zhang:** Methodology. **Hongyang Lu:** Investigation. **Wei He:** Software, Resources. **Li Yu:** Methodology. **Quazi T.H. Shubhra:** Writing – review & editing. **Changlong Xu:** Funding acquisition. **Xiaojun Cai:** Conceptualization, Funding acquisition, Supervision, Writing – review & editing.

## Declaration of competing interest

The authors declare that they have no known competing financial interests or personal relationships that could have appeared to influence the work reported in this paper.
