# Supplementary material for: CO-mediated restoration of mitochondrial redox homeostasis contributes to alleviating multiple programmed cell deaths in hepatic I/R injury
Source: Redox Biol. 2026 Oct 15;98:104440. doi: 10.1016/j.redox.2026.104440 (PMC13692684; doi:10.1016/j.redox.2026.104440)
Supplement: Supplementary file 1 [file mmc1.docx]

**CO‑mediated restoration of mitochondrial redox homeostasis contributes to alleviating multiple programmed cell deaths in hepatic I/R injury**

Jiayu Li^a,†^, Mengzhu Xu^a,†^, Qiang Zhou^b,*^, Hanjing Ni^c^, Chengge Shi^a^, Tianci Zuo^a^, Lingjun Zhang^a^, Hongyang Lu^a^, Wei He^a^, Li Yu^a^, Quazi T.H. Shubhra^d^, Changlong Xu^c,^*，Xiaojun Cai^a,^*

*^a^*School and Hospital of Stomatology, Wenzhou Medical University, Wenzhou 325027, China

*^b^*Department of Neurosurgery, Shanghai Tenth People's Hospital of Tongji University, Shanghai 200072, China.

*^c^*Department of Gastroenterology, the Second Affiliated Hospital and Yuying Children's Hospital of Wenzhou Medical University, Wenzhou 325024, China

*^d^*Institute of Chemistry, University of Silesia in Katowice, Szkolna 9, Katowice 40-006, Poland

*Corresponding author. Tel.: +8618068834513; fax: +86 0577 88855488.

E-mail addresses: [zqreadmore@163.com](mailto:zqreadmore@163.com); [xchlong@163.com](mailto:xchlong@163.com); [cxj520118@njtech.edu.cn](mailto:cxj520118@njtech.edu.cn) (Xiaojun Cai); [cxj520118@wmu.edu.cn](mailto:cxj520118@wmu.edu.cn) (Xiaojun Cai).

^†^These authors made equal contributions to this work.

**Fig. S1** Representative photographs showing the color change of CPs@HA and CPs solutions during 7 days of storage at 37 °C.

**Fig. S2** Particle size variations of CPs@HA and CPs in PBS solution containing 15% FBS over 48 h of incubation.

**Fig. S3** Zeta potential variations of CPs@HA and CPs in PBS solution containing 15% FBS over 48 h of incubation.

**Fig. S4** UV–vis absorption spectra of CORM-401, CPs, CPs@HA, PAMAMs, and HA in PBS solution.

**Fig. S5** Standard calibration curve of CORM-401 in a mixed solution of methanol and PBS (methanol: PBS = 1:21).

**Fig. S6** CO release from free CORM-401 and CPs@HA in the presence of 1 mM H₂O₂.

**Fig. S7** Size distribution and solution appearance (inset) of iCPs@HA

**Fig. S8** *In vitro* cytocompatibility of CPs@HA. Representative live/dead staining images of RAW264.7 cells after different treatments.

**Fig. S9** *In vitro* evaluation of RAW264.7 cell viability after different treatments. Statistical analysis was performed by comparing the Ctrl group with each of the other groups (n = 6); *p < 0.05, **p < 0.01, ***p < 0.001, and ns indicates no significant difference.

**Fig. S10** *In vitro* representative live/dead staining images of HepG2 cells after different treatments.

**Fig. S11** *In vitro* cell viability of HepG2 cells after different treatments. Statistical analysis was performed by comparing the Ctrl group with each of the other groups (n = 6); *p < 0.05, **p < 0.01, ***p < 0.001, and ns indicates no significant difference.

**Fig. S12** *In vitro* hemolysis assay results and representative photographs of red blood cell suspensions after different treatments (n = 6); *p < 0.05, **p < 0.01, ***p < 0.001, and ns indicates no significant difference.

**Fig. S13** *In vitro* fluorescence intensity of Cy5.5 in LPS-induced activated macrophages treated with Cy5.5-CPs and Cy5.5-CPs@HA, indicating the cellular uptake efficiency of nanogels. Statistical analysis was performed by comparing the CPs@HA (150 μg/mL) group with each of the other groups (n = 3); ***p < 0.001.

**Fig. S14** *In vitro* fluorescence intensity of Cy5.5 in LPS-induced HepG2 cells treated with Cy5.5-CPs and Cy5.5-CPs@HA, indicating the cellular uptake efficiency of nanogels. Statistical analysis was performed by comparing the CPs@HA (150 μg/mL) group with each of the other groups (n = 3); ***p < 0.001.

**Fig. S15** *In vitro* intracellular fluorescence intensity of the CO probe in LPS-induced activated macrophages. Statistical analysis was performed by comparing the CPs@HA (150 μg/mL) group with each of the other groups (n = 3); ***p < 0.001.

**Fig. S16** *In vitro* intracellular fluorescence intensity of the ROS probe in LPS-induced activated macrophages. Statistical analysis was performed by comparing the LPS group with each of the other groups (n = 3); ***p < 0.001.

**Fig. S17** *In vitro* intracellular fluorescence intensity of the H_2_O_2_ probe in LPS-induced activated macrophages. Statistical analysis was performed by comparing the LPS group with each of the other groups (n = 3); ***p < 0.001.

**Fig. S18** *In vitro* bright-field images of RAW264.7 cell morphology after different treatments.

**Fig. S19** *In vitro* quantification of RAW264.7 cell elongation after different treatments. Statistical analysis was performed by comparing the LPS group with each of the other groups (n = 3); *p < 0.05, ***p < 0.001, and ns indicates no significant difference.

**Fig. S20** *In vitro* quantification of RAW264.7 cell protrusions after different treatments. Statistical analysis was performed by comparing the LPS group with each of the other groups (n = 3); *p < 0.05, ***p < 0.001, and ns indicates no significant difference.

**Fig. S21** *In vivo* dose evaluation CPs@HA in the mouse HIRI model. Serum levels of (**a**) ALT, (**b**) AST, and (**c**) COHb in HIRI mice treated with different concentrations of CPs@HA. Statistical analysis was performed by comparing the HIRI group with each of the other groups (n = 3); **p < 0.01, ***p < 0.001, and ns indicates no significant difference.

**Figure S22** Schematic overview of the *in vivo* experimental design and analyses (created with BioRender.com)

**Fig. S23** *Ex vivo* fluorescence imaging of isolated organs from HIRI mice treated with Cy5.5-CPs@HA and Cy5.5-CPs at 24 h post-injection, and the corresponding Cy5.5 fluorescence intensity in each organ. Statistical analysis was performed by comparing the Cy5.5 fluorescence intensity between the Cy5.5-CPs@HA and the Cy5.5-CPs in the same organ (n = 3); **p < 0.01, ***p < 0.001, and ns indicates no significant difference.

**Fig. S24** Representative fluorescence images of the CO probe (green) in frozen liver sections from the *in vivo* mouse HIRI model following different treatments, indicating CO release from different nanogels at the HIRI site.

**Fig. S25** Quantification of CO probe fluorescence intensity in frozen liver sections from the *in vivo* mouse HIRI model. Statistical analysis was performed by comparing the CPs@HA group with each of the other groups (n = 3); ***p < 0.001.

**Fig. S26** *In vivo* dynamic changes of serum COHb levels within 24 h after intravenous injection. Statistical analysis was performed by comparing COHb levels between the Normal group and other groups at different time points (n = 3); ns indicates no significant difference.

**Fig. S27** *In vivo* complete blood count parameters (Hb, RBC, PLT, WBC, NEUT) in mice of the Normal, HIRI, and CPs@HA groups at 24 h post-injection. Statistical analysis was performed by comparing the Normal group with each of the other groups (n = 3); ***p < 0.001, ns indicates no significant difference.

**Fig. S28** *In vivo* representative H&E staining images of major organs (heart, liver, spleen, lung, and kidney) from mice in the Normal and CPs@HA groups.

**Fig. S29** *In vivo* quantification of 8-OHdG fluorescence intensity in liver tissues. Statistical analysis was performed by comparing the HIRI group with each of the other groups (n = 3); **p < 0.01, ***p < 0.001, and ns indicates no significant difference.

**Fig. S30** *In vivo* CAT activity in liver tissues. Statistical analysis was performed by comparing the HIRI group with each of the other groups (n = 3); **p < 0.01, and ns indicates no significant difference.

**Fig. S31** *In vivo* quantification of CD86 fluorescence intensity in liver tissues. Statistical analysis was performed by comparing the HIRI group with each of the other groups (n = 3); ***p < 0.001, and ns indicates no significant difference.

**Fig. S32** *In vivo* quantification of CD206 fluorescence intensity in liver tissues. Statistical analysis was performed by comparing the HIRI group with each of the other groups (n = 3); *p < 0.05, ***p < 0.001, and ns indicates no significant difference.

**Fig. S33** *In vivo* quantification of GPX4 fluorescence intensity in liver tissues. Statistical analysis was performed by comparing the HIRI group with each of the other groups (n = 3); *p < 0.05, ***p < 0.001, and ns indicates no significant difference.

**Fig. S34** *In vivo* auantification of DLAT fluorescence intensity in liver tissues. Statistical analysis was performed by comparing the HIRI group with each of the other groups (n = 3); **p < 0.01, ***p < 0.001, and ns indicates no significant difference.

**Fig. S35** Schematic overview of the in vitro treatment and cell death assays (created with BioRender.com).

**Fig. S36** *In vitro* establishment of an RSL3-induced ferroptosis model in HepG2 cells. Cell viability of HepG2 cells treated with different concentrations of RSL3. Statistical analysis was performed by comparing the viability of cells treated with 64 μg/mL RSL3 with each of the other groups (n = 3); ***p < 0.001.

**Fig. S37** *In vitro* protective effect of CPs@HA against RSL3‑induced ferroptosis in HepG2 cells as determined by cell viability assay. Statistical analysis was performed by comparing the RSL3 group with each of the other groups (n = 3); ***p < 0.001.

**Fig. S38** *In vitro* protective effect of CPs@HA against RSL3‑induced ferroptosis in HepG2 cells as determined by live/dead staining.

**Fig. S39** *In vitro* quantification of ROS levels in HepG2 cells subjected to RSL3‑induced ferroptosis, with or without CPs@HA treatment.

**Fig. S40** *In vitro* quantification of GSSG content and GSH/GSSG ratio in HepG2 cells subjected to RSL3 induced ferroptosis, with or without CPs@HA treatment. Statistical analysis was performed by comparing the RSL3 group with each of the other groups (n = 3); **p < 0.01, ***p < 0.001, and ns indicates no significant difference.

**Fig. S41** *In vitro* cell viability of HepG2 cells treated with different concentrations of CuCl_2_.

**Fig. S42** *In vitro* cell viability of HepG2 cells treated with different concentrations of ES.

**Fig. S43** *In vitro* cell viability of HepG2 cells treated with different concentrations of CuCl_2_ in the presence of 100 nM ES.

**Fig. S44** *In vitro* protective effect of CPs@HA against CuCl_2_/ES‑induced cuproptosis in HepG2 cells as determined by cell viability assay. Statistical analysis was performed by comparing the CuCl_2_/ES group with each of the other groups (n = 3); ***p < 0.001.

**Fig. S45** *In vitro* protective effect of CPs@HA against CuCl_2_/ES‑induced cuproptosis in HepG2 cells as determined by live/dead staining.

**Fig. S46** *In vitro* quantification of DLAT fluorescence intensity in HepG2 cells subjected to CuCl_2_/ES‑induced cuproptosis, with or without CPs@HA treatment. Statistical analysis was performed by comparing the CuCl_2_/ES group with each of the other groups (n = 3); ***p < 0.001.

**Fig. S47** *In vitro* quantification of GSSG content in HepG2 cells subjected to CuCl_2_/ES‑induced cuproptosis, with or without CPs@HA treatment. Statistical analysis was performed by comparing the CuCl_2_/ES group with each of the other groups (n = 3); **p < 0.01.

**Fig. S48** *In vitro* cell viability of HepG2 cells upon receiving different durations of glucose starvation treatment.

**Fig. S49** *In vitro* protective effect of CPs@HA against glucose starvation‑induced disulfidptosis in HepG2 cells as determined by cell viability assay. Statistical analysis was performed by comparing the Glu(-) group with each of the other groups (n = 3); ***p < 0.001.

**Fig. S50** Representative line-scan profiles of MitoTracker and Rhod-2 fluorescence intensities in liver tissues from from the *in vivo* mouse HIRI model after different treatment.

**Table S1** CORM-401 loading content and loading efficiency of CPs at different feeding ratios of CORM-401 to PAMAMs.

|  | CORM401/PAMAMs  (*w*/*w*) | CORM 401  LC (%) | CORM 401  LE (%) |
| --- | --- | --- | --- |
| CPs | 0.5/1 | 10.27 | 65.62 |
| CPs | 1/1 | 18.60 | 62.00 |
| CPs | 2/1 | 19.10 | 36.53 |

**Table S2** CORM-401 loading content and loading efficiency of CPs@HA at different feeding ratios of CPs to HA; the CPs were prepared at a feeding ratio of CORM-401 to PAMAMs (1/1).

|  | CPs/ HA  (*w*/*w*) | CORM 401  LC (%) | CORM 401  LE (%) |
| --- | --- | --- | --- |
| CPs@HA | 0.5/1 | 8.59 | 61.71 |
| CPs@HA | 1/1 | 14.45 | 57.80 |
| CPs@HA | 2/1 | 14.87 | 31.90 |

**Table S3** Characteristic gene sets of programmed cell death pathways used for ssGSEA analysis.

| Pathways | Factor positive | Factor negative |
| --- | --- | --- |
| Ferroptosis | ACSL4, NCOA4, SAT1, TFRC, ALOX15, LPCAT3, NOX1, STEAP3, SLC11A2 | GPX4, SLC7A11, FTH1, FTL GCLM, GSS, SLC3A2 |
| Disulfidptosis | SLC7A11, NCKAP1, RPN1, CYB5R1, RAC1, ACTB | G6PD, PRDX1, TXNRD1, GLRX, SOD1, CAT, MTF1 |
| Cuproptosis | FDX1, LIAS, DLD, PDHA1, PDHB, DLAT, DBT, GCSH | MTF1, CDKN2a, GLRX5, NFE2l1, NFE2l2, ATOX1, COX17 |
| Sodioptosis | SCNN1A, SCNN1B, SCNN1G, SCN1A, SCN2A, SCN3A, SCN8A, SLC9A1, SLC9A2, SLC9A3, SLC12A1, SLC12A2, SLC12A3, SLC5A1, SLC5A2, SLC5A3, AQP1, AQP3, AQP5, TRPV4, TRPM7, SLC8A1, SLC8A2, SLC8A3, ATP1A1, ATP1A2, ATP1B1 | HMOX1, NFE2L2, FXYD2, FXYD3, FXYD7, KCNJ2, KCNJ10, KCNA1, KCNQ1, KCNQ2, KCNQ3, TMEM206, CLCN3, BEST1, SGK1, HSP90AA1, HSPA1A, HSPA8 |
| Parthanatos | PARP1, PARP2, AIFM1, MIF, H2AFX, XRCC1, XRCC5, XRCC6, NEIL1, NEIL2, PARPBP, NAMPT, NMRK1, NMRK2, SARM1, NOS2, CYBB, NOX1, NOX4, ENDOG, DFFB | PARP3, PARP9, NMNAT1, NMNAT2, NMNAT3, QPRT, IDH1, IDH2, MDH1, PPARGC1A, PPARGC1B, GPX4, PRDX1, SOD2, BCL2, BCL2L1, CYBA |

**Table S4** Death potential index scores of programmed cell death pathways across different samples.

| Sample | FPI | CPI | DPI | PPI | SPI |
| --- | --- | --- | --- | --- | --- |
| A1 | -0.211881725 | -0.824054 | 0.0789837077888037 | -0.2089 | -0.489313 |
| A2 | -1.127652459 | -0.800286 | -0.943601 | 0.405322854188778 | 0.168254493234879 |
| A3 | -1.088574238 | -0.4915 | -0.914431 | 0.0692425935818124 | -0.402652 |
| A4 | -1.188449167 | -0.474379 | -0.8271 | 0.497028114889901 | -0.309691 |
| A5 | -0.66986812 | -0.812855 | -1.193896 | -0.316179 | -0.185843 |
| B1 | 0.560636396005706 | 1.08310538274283 | 0.251347802727144 | -0.325492 | 0.63005335411477 |
| B2 | 0.760495532342746 | 1.48342718875509 | 1.29421312866826 | -0.140626 | 0.329084543108174 |
| B3 | 1.16906520222755 | 0.520448897148516 | 1.28001716826794 | 0.0647583664289078 | 0.659556199817058 |
| B4 | 0.918900111000426 | 0.673891666472424 | 0.748723368713103 | 0.145411212980256 | 0.525657877337483 |
| B5 | 0.725057047700564 | 0.694627212879051 | 0.740285342637721 | -0.018222 | 0.136162547287226 |
| C1 | 0.196127014130906 | -0.082362 | 0.607002358805007 | 0.0319536286951368 | -0.121706 |
| C2 | -0.241777302 | -0.731766 | 0.297204424440757 | -0.024553 | -0.205274 |
| C3 | -0.176777789 | -1.116342 | -0.830716 | 0.19343425257997 | 0.178043470649213 |
| C4 | -0.108284091 | 0.213325272508307 | -0.523343 | -0.52398 | -0.69032 |
| C5 | 0.074465698207279 | -0.333396 | -0.833578 | -0.229532 | -0.654818 |

**Table S5** Primer sequences of genes used in the study.

| Genes | Forward primer sequence (5′–3′) | Reverse primer sequence (3′–5′) |
| --- | --- | --- |
| HO-1 | AAGACTGCGTTCCTGCTCAAC | AAAGCCCTACAGCAACTGTCG |
| TNF-α | TCCAGGCGGTGCCTATGTC | CCTCCACTTGGTGGTTTGTGA |
| IL-1β | TGGAGAGTGTGGATCCCAAG | GGTGCTGATGTACCAGTTGG |
| iNOS | CGCTTGGGTCTTGTTCACT | TCTTCAGGTCACTTGGTA |
| CD206 | GGACGAAAGGCGGGATGT | GGGTTCAGGAGTTGTTGTGGG |
| Arg-1 | GTGAACACGGCAGTGGCTTTA | AGGCGTTTGCTTAGTTCTGTCTG |
| IL-4 | GTCATCCTGCTCTTCTTTCTCGA | TGAAGGTATCAGCGGAGGGA |
| GAPDH (RAW 264.7) | ACCCTTAAGAGGGATGCTGC | CATCAGCAGCAGCCATGTACTCTTCAC |
